# Graphene-based materials: an innovative approach for neural regeneration and spinal cord injury repair

**DOI:** 10.1039/d4ra07976k

**Published:** 2025-03-31

**Authors:** Ayda Yari-Ilkhchi, Nazila Hamidi, Mehrdad Mahkam, Abbas Ebrahimi-Kalan

**Affiliations:** a Chemistry Department, Faculty of Science, Azarbaijan Shahid Madani University 5375171379 Tabriz Iran ayda.yari90@gmail.com; b Faculty of Chemical and Metallurgical Engineering, Department of Chemical Engineering, Istanbul Technical University Maslak 34469 Istanbul Turkey; c Faculty of Engineering and Natural Sciences, Sabanci University 34956 Istanbul Turkey; d Department of Chemistry and Biochemistry, The University of Tulsa Tulsa OK 74104 USA; e Faculty of Advanced Medical Science, Tabriz University of Medical Sciences 5166614733 Tabriz Iran abbasebra@gmail.com Ebrahimiab@tbzmed.ac.ir

## Abstract

Spinal cord injury (SCI), the most serious disease affecting the central nervous system (CNS), is one of contemporary medicine's most difficult challenges, causing patients to suffer physically, emotionally, and socially. However, due to recent advances in medical science and biomaterials, graphene-based materials (GBMs) have tremendous potential in SCI therapy due to their wonderful and valuable properties, such as physicochemical properties, extraordinary electrical conductivity, distinct morphology, and high mechanical strength. This review discusses SCI pathology and GBM characteristics, as well as recent *in vitro* and *in vivo* findings on graphenic scaffolds, electrodes, and injectable achievements for SCI improvement using neuroprotective and neuroregenerative techniques to improve neural structural and functional repair. Additionally, it suggests possible ideas and desirable products for graphene-based technological advances, intending to reach therapeutic importance for SCI.

## Abstract 2

This review explores the pathology of SCI and the characteristics of GBMs, focusing on recent *in vitro* and *in vivo* research on their mechanism, biocompatibility, toxicity, and biodegradability.

## Graphical abstract

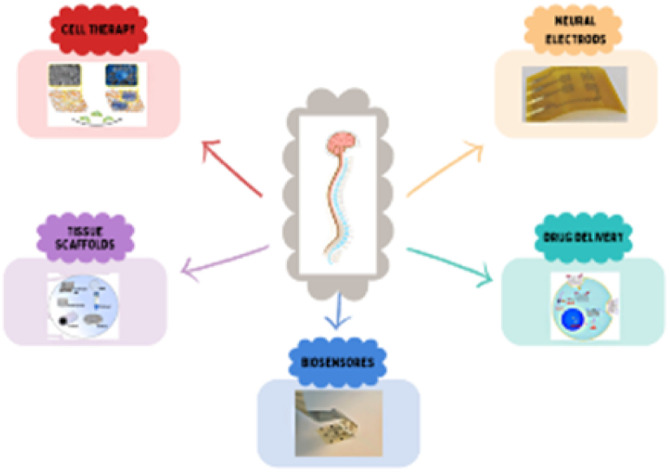

## 1 Introduction

The human nervous system consists of two parts: the central nervous system (CNS), which contains the spinal cord and the brain, and the peripheral nervous system (PNS), which includes the ganglia and nerves that surround the CNS. Nervous system lesions are generally difficult and serious problems all over the world. While acute PNS injuries often heal spontaneously, CNS tissue lesions do not heal as rapidly. Spinal cord injuries (SCIs), a subset of CNS damage, depend on minimizing secondary injuries through physical therapy. Various researchers worldwide are investigating the treatment and regeneration of injured tissue, as well as the return of sensory and motor functions.^1–4^

Damage to the CNS is generally more severe and debilitating than harm to the PNS, making recovery more challenging.^5,6^ Renovation and recovery of nervous system lesions have been challenging compared with other tissues because of their function and anatomy. SCI impacts thousands of individuals worldwide each year, leading to significant medical expenses and severe repercussions such as sensory loss, paralysis, and bowel/bladder dysfunctions. According to the World Health Organization, annual morbidity is 40–80 cases per million worldwide.^2,7,8^ Between 1990 and 2016, nearly 27 million people have suffered from SCI throughout the world.^9^ Alarmingly, 2019 saw an estimated 9 million new cases of SCI, marking a 52.7% increase compared to 1990 projections.^10^

In the CNS system, particularly in SCI, an important factor inhibiting nerve regeneration is the formation of thick glial scar tissue by astrocytes. Besides, meningeal cell activity and the absence of Schwann cells decline axonal growth.^1,11,12^ There are high hopes for advancing research on the restoration and stimulation of nervous system lesions by bionanomaterials. Bionanomaterials significantly enhance astrocyte activity by providing supportive scaffolds for adhesion and growth, releasing bioactive molecules to modulate behavior, and delivering electrical and mechanical stimuli to promote beneficial responses.^13–15^ These materials also facilitate interactions between astrocytes and neurons, improving synaptic plasticity and neural network function.^13^

Graphene-based materials (GBMs), due to their unique electrical, mechanical, and physicochemical properties, are suggested as promising candidates for nerve stimulation and regeneration. Moreover, modification of GBMs with biocompatible compounds generates several surface charges and can affect the length of neurite outgrowth, their number, branching, and the number of synaptic junctions.^16–18^

This review summarizes and discusses the most recent investigations on the utilization of GBMs in neural cell activities, differentiation, proliferation, and the regeneration of nerves, as well as the toxicity and biocompatibility of these compounds targeting SCI treatment.

## 2 Physiopathology of SCI

After damaging the spinal cord, a cascade of interactions leads to neurological deficits. In the acute phase, ionic imbalance promotes calcium invasion, causing cell death *via* mitochondrial failure and the creation of free radicals. Glutamate accumulation increases excitotoxicity. Inflammatory cells cross the blood-spinal cord barrier (BSB), intensifying tissue damage and expanding dysfunction. Neuroinflammation develops when macrophages and microglia switch between inflammatory (M1) and anti-inflammatory/regenerative (M2) phenotypes. Both phenotypes initially exist, although M2 preponderance decreases over time. Astrocyte proliferation and scar formation indicate astrogliosis, which isolates the injury site. Astrocytes cause inflammation and scar formation and change the microenvironment. Scar tissue, made up of astrocytes, fibroblasts, and immune cells, is critical in SCI and increases the lesion site. For inducing reparative responses, the interaction between neurites and immune cells is essential, with diverse macrophage populations mediating responses from neurotoxic to neuroregenerative.^19,20^ In the acute phase of SCI, necrosis, ischemia, and inflammation in the injured tissue directly compromise the immune system. In the chronic phase, other pathogenic agents, such as dysregulation mediated by dysautonomia or gut dysbiosis, further challenge the immune system.^7,21^

**Fig. 1:**
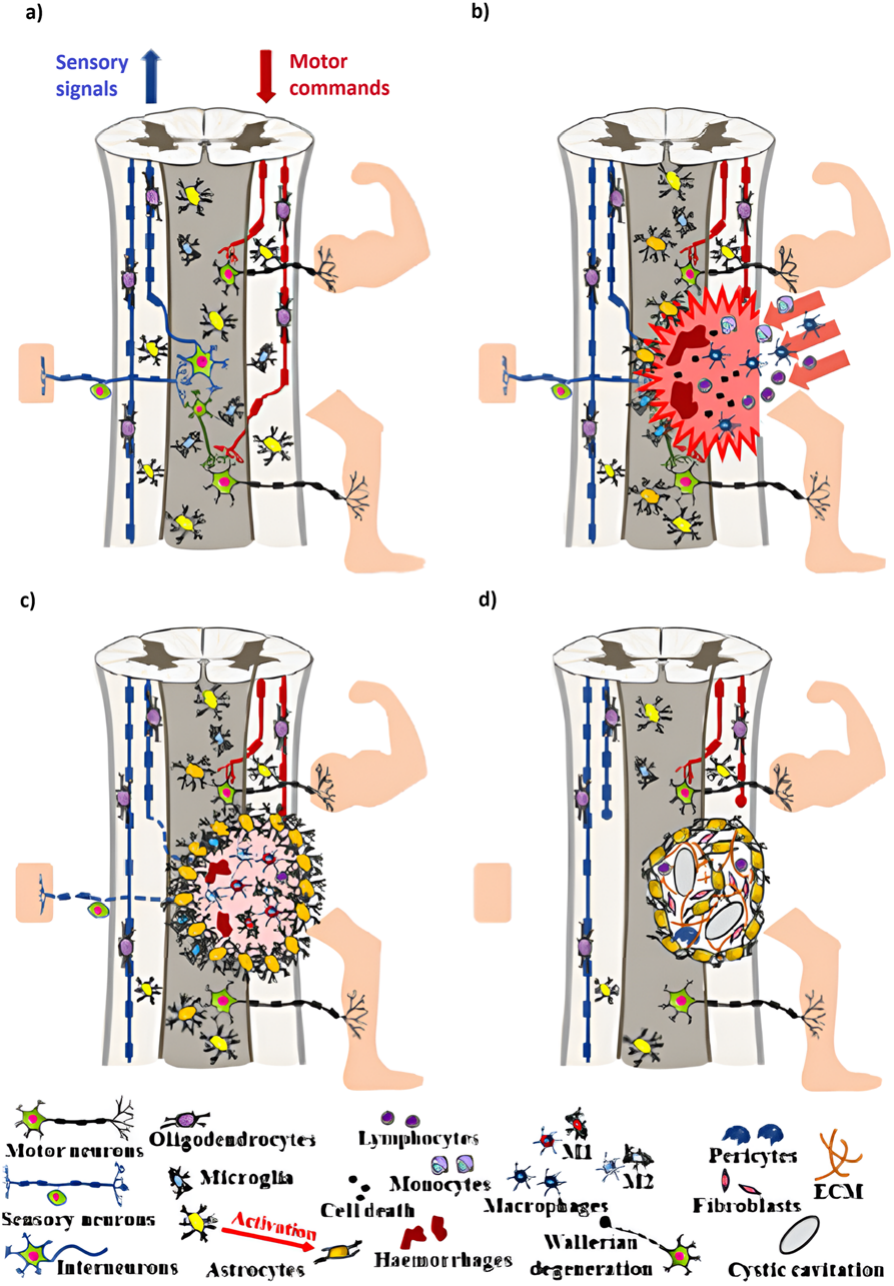
Pathophysiology of a Traumatic Spinal Cord Injury (TSCI). (a) Healthy spinal cord schematic: ascending pathways provide somatic sensory information from receptors to the brain and upper spinal cord *via* sensory neurons. In contrast, descending tracts transmit information from the cerebral cortex to motor neurons. (b) The acute phase of TSCI is characterized by cell death and neural circuit disruption. (c) Subacute phase of TSCI: the inflammatory cascade during this stage promotes cell death and demyelination. (d) Intermediate and chronic stages of TSCI: astrogliosis causes the creation of a spinal injury scar, which has both protective and inhibitory roles.^5^ Copyright 2022, *ACS Nano* with the CC-BY4.0 license.

Currently, treating SCI is a significant difficulty for both researchers and physicians. Two primary theories influence the absence of useful treatments: (i) the movement system of the human spinal cord depends more relatively on supraspinal control compared to other mammals; and (ii) SCI causes dormancy in spinal circuitry, reducing its ability to generate responses.^2^ Other negative associated factors that limit natural regeneration ability of the central nervous tissue include the swift formation and increase of inhibitory fibroglial scars (Fig. 1).

## 3 Biomaterials in regeneration of nerves system and SCI

SCI results from both primary and secondary injuries involving inflammation, vascular changes, and more, leading to additional neuronal damage. The injury disrupts spinal cord white matter, which contains tracts of axons that transmit signals to and from the brain. Current treatments, including surgical decompression and methylprednisolone, are limited in their effectiveness. Unlike peripheral neurons, spinal cord axons have limited regenerative capacity, often leading to cystic cavities and a lack of growth-permissive substrates. Additionally, inhibitory molecules like myelin-associated proteins, chondroitin sulfate proteoglycans, and physical barriers like glial scars impede regeneration.^22,23^ Despite these challenges, some research indicates potential avenues for regeneration, such as manipulating specific genes and using neural stem cells. Axonal regeneration in the spinal nervous system requires the interaction of cell membrane receptors called integrins with extracellular matrix (ECM) components. Integrins, highly expressed in the developing nervous system, are downregulated in adult neurons but play a crucial role in the growth cones of regenerating axons. Important ECM molecules like collagens, laminins, and fibronectin bind to integrin receptors to support axonal regeneration. The presence of insufficient physical and molecular substrates for axonal attachment in the injured site of spinal cord, making it difficult for axons to grow into cystic lesion sites. Biomaterials designed to mimic ECM architecture can help provide the necessary substrates for axon attachment and facilitate regeneration.^22,24^

Biomaterials are generally categorized into two types: natural and synthetic. Natural biomaterials can be constructed from ECM proteins. For instance, collagen and laminin hydrogels have been used in various tissue engineering applications, including nervous system injuries and SCI. ECM-based biomaterials typically integrate well into injured tissue and support axonal outgrowth.^23,24^ However, ECM hydrogels often degrade quickly *in vivo* and have low mechanical strength. Natural biomaterials also include non-ECM materials, such as alginate, derived from algae, and chitosan, obtained from the outer skeleton of shellfish. Their degradation period can be adjusted to meet specific needs, but they are composed of non-permissive molecules and cannot specifically interact with mammalian cells. Synthetic biomaterials include polylactic acid (PLA), poly lactic-*co*-glycolic acid (PLGA), polycaprolactone (PCL), polyethylene glycol (PEG), and several others. These materials can generally be more easily modified than natural biomaterials. For example, their porosity, rigidity, and degradation rate can be tailored to match different tissue types. However, synthetic biomaterials may require ECM coating or other surface modifications since they do not contain integrin-binding molecules.^22–25^ Biomaterials, such as scaffolds and nanoparticles, have gained significant attention for their roles in neuroprotection and neuroregeneration.^26–28^

**Fig. 2:**
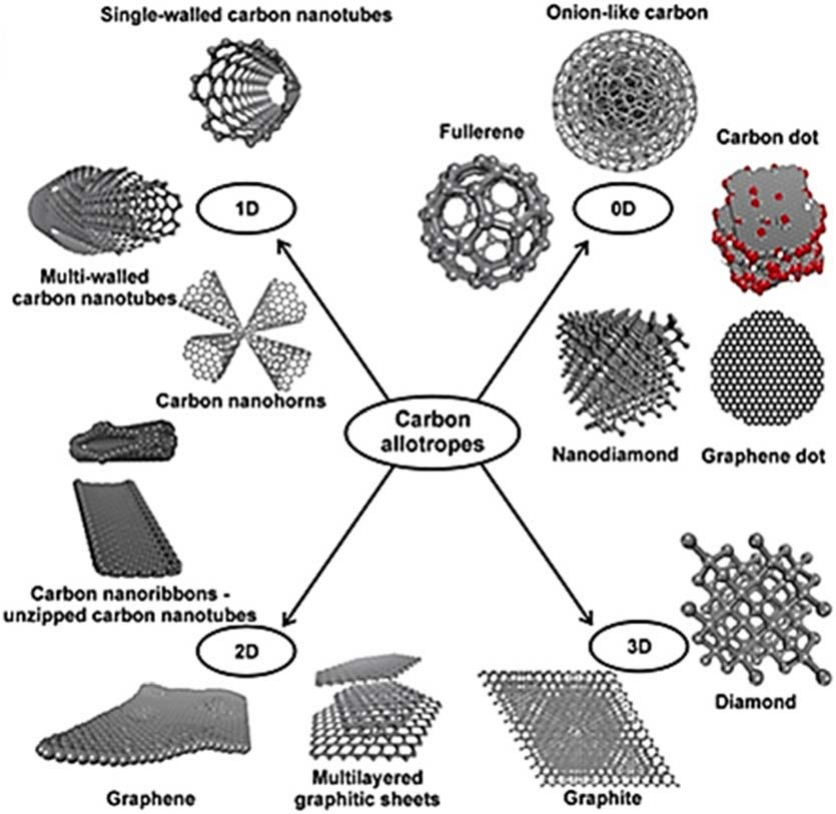
Classification of carbon allotropes. Reproduced with permission.^25^ Copyright Elsevier 2020.

Nanoparticles, due to their unique physicochemical properties, show promising potential in neural engineering and other technological applications. They effectively deliver therapeutics into cells, enabling controlled, targeted delivery for better healthcare treatments. Currently, nanoparticles are being explored for treating nerve injuries, with several commercialized products already available, and they have the potential to prevent or treat neurodegeneration by manipulating their properties.^24^ These materials are classified into two main categories: inorganic and organic. Inorganic nanomaterials consist of metals, alloys, silica, magnetic materials, and quantum dots, while various organic nanomaterials include liposomes, micelles, dendrimers, polymeric nanoparticles, and nanofibers, as well as carbon-based nanomaterials.^24,25^ Carbon-based nanomaterials (CNMs), including carbon nanotubes (CNTs), graphene, and fullerenes, contain sp^2^-bonded carbon and are classified based on dimensionality: zero-dimensional (fullerene, 1985), one-dimensional (CNTs, 1991), and two-dimensional (graphene, 2004). These materials are attractive for various applications due to their unique physicochemical, optical, thermal, mechanical, and electrical properties and have been studied in biomedical applications, showing promise for neuro-engineering. However, their application is limited by cytotoxicity issues, which depend on size, thickness, concentration, and preparation process. Fig. 2 illustrates various classifications of carbon allotropes.^25^

## 4 Introduction, chemistry, and structural classification of GBMs

Graphene, a two-dimensional honeycomb lattice, is one of the magic materials. This one-atom-thick carbon system is the world's thinnest and most active substance. It is an excellent conductor, having a large surface area, high carrier transport volubility, high flexibility, and unique thermal-chemical properties. Due to these unique characteristics, graphene has been highly utilized in multiple areas and is extremely desired in nanomedicine, containing antiviral, antibacterial, and parasitical applications, gene delivery, drug delivery, ultra-sensitive biosensing (*e.g.*, DNA detection), early detection of diseases (*e.g.*, early diagnosis of leukemia), regenerative medicine, targeting, monitoring, treatment of cancer cells, and tissue engineering based on stem cells.^29–33^

Graphene has emerged as a promising candidate for repairing injured nerves. It can be synthesized through both top–down and bottom–up approaches. Despite its advantages over other CNMs, graphene has limitations, such as an unstable chemical structure and insufficient active sites, which hinder its interaction with other biomolecules. To address these limitations, chemical modification of graphene is highly recommended.^17,35^ GBMs can be categorized into several distinct classes based on their structural characteristics, intrinsic properties, and synthesis methodologies.^34,35^

Generally, GBMs, based on their structural forms, consist of single-layer and multi-layer graphene, graphene oxide (GO), reduced graphene oxide (rGO), graphene oxide quantum dots (GOQDs), and graphene quantum dots (GQDs). These materials exhibit several chemical and physical properties, such as variation in number of layers, surface chemistry, low density, conductivity, and mechanical properties.^36–39^

Single-layer graphene, a pristine and unmodified form of graphene, is known for its exceptional electrical conductivity and mechanical strength.^17^ Graphene was discovered through a scotch tape peeling process in 2004, generating massive interest throughout scientific associations.^40^ Currently, various cutting-edge technologies are used to produce graphene. Graphene and its derivatives have been prepared using various methods, including chemical vapor deposition (CVD),^41^ liquid-phase exfoliation,^42,43^ micromechanical exfoliation,^44^ and chemical^45^/electrochemical exfoliation.^46^

It is mainly used in basic research to understand the fundamental interactions with neural cells and to explore its potential for neural tissue engineering.^9^

Multi-layer graphene, composed of multiple graphene layers held together by weak van der Waals forces, maintains several advantageous properties of single-layer graphene. However, the mechanical integrity of multi-layer graphene can be affected by potential slippage between layers. It is frequently employed in composite materials where precise electronic structuring is less crucial compared to single-layer applications.^47,48^

GO, is a derivative of graphene with an enhanced capacity to absorb biomolecules. This is due to the presence of different types of oxygen-containing functional groups on the surface of the GO basal sheet, including carboxyl groups (–COOH) at the edges, as well as epoxy (–O) and hydroxyl (–OH) groups on the basal plane.^17,34,49^ This allows GO to functionalize in a non-covalent, covalent, and/or ionic manner by interacting with an extensive range of organic and inorganic materials to produce a variety of hybrids and composites.^50–52^ GO is synthesized using both the Hummers and Offeman method *via* oxidative exfoliation of graphite as early as the 1950s. In this method, graphite reacts with a mixture of concentrated sulfuric acid (H_2_SO_4_) and potassium permanganate (KMnO_4_), oxidizing incredibly with large numbers of hydroxyl, epoxide, and carboxyl functional groups on their surfaces.^53–56^

GO exhibits electronically hybrid characteristics due to the conducting π-states from sp^2^ carbon sites and a significant energy gap among the σ-states of its sp^3^ bonded carbons. This hybrid character is caused by the presence of sp^2^ and sp^3^ hybridized carbon atoms, which can be altered throughout the chemical reduction process. Tuning the ratio of sp^2^ to sp^3^ areas can influence GO's bandgap, changing it from an insulator to a semiconductor. GO undergoes chemical or thermal reduction, resulting in the loss of CO or CO_2_ from the basal plane. This reduction process seeks to obtain graphene-like characteristics; however, residual defects such as oxygen atoms, holes, and Stone–Wales defects (pentagon–heptagon pairs) may remain.^57–59^ GO promotes neural stem cell (NSC) adhesion, proliferation, and differentiation, serves as a scaffold for drug delivery, and enhances the survival of NSCs *in vitro* and *in vivo*.^9^

rGO, a notable member of the graphene family, is produced by chemically reducing the oxygen content in GO through thermal, chemical, or UV exposure processes.^17,48^ This results in improved electrical conductivity and mechanical properties compared to GO. rGO is hydrophobic and is commonly used as a nanofiller or for coating medical devices.^34,49^ It is used in scaffolds to promote axonal growth and neuronal differentiation and enhances electrical stimulation of neural cells, which can be beneficial for recovery after SCI.^9^

GQDs are nanoscale graphene fragments, under 10 nanometers in size, with unique optical and electronic properties due to quantum confinement effects. They can be synthesized *via* top–down methods like chemical oxidation and laser ablation, or bottom–up techniques such as CVD and hydrothermal synthesis, allowing control over their size, surface functionalization, and properties.^60^ Their high sp^2^ hybridization contributes to excellent electronic properties, and modifying their size and surface chemistry allows tunable optical characteristics like fluorescence. In biomedicine, GQDs are promising for drug delivery, imaging as fluorescent probes, and biosensing due to their high sensitivity and specificity. For the CNS, GQDs show neuroprotective effects and can cross the blood–brain barrier (BBB), suitable for neuroimaging and targeted drug delivery in neurological disorders. Research suggests GQDs can interact with neural cells, potentially aiding in treating conditions like Alzheimer's by obstructing amyloid-beta peptide aggregation linked to neurodegeneration.^60–62^

### 4.1 Functionalization of GBMs

Functionalizing graphene entails the modification of its structure through chemical, physical, or hybrid methods to enhance existing properties or introduce new functionalities. This process aims to improve the material's manipulability and tailor it to meet specific application requirements. The products resulting from graphene functionalization are referred to as functionalized graphene materials, which can be further categorized into functionalized graphene and functionalized graphene composites.^63^ Despite the significant potential of pristine graphene, its applications are limited compared to more established materials due to its lack of a band gap, low chemical reactivity, and poor water dispersibility. However, these limitations can be overcome through the functionalization of GBMs. Graphene derivatives, like GO and rGO, can be altered with a variety of organic or inorganic molecules *via* chemical or physical methods.^48^ The incorporation of various oxygen-containing groups and sp^2^ domains allows these materials to interact with other molecules through covalent, non-covalent, or a combination of interactions. This results in hybrid or composite materials that exhibit enhanced properties such as improved dispersibility, processability, purification, device fabrication, biocompatibility, and modifications to the band gap. While these general trends are not strict rules, they can be beneficial depending on the specific materials being used for immobilization.^62,63^ This improves specificity and efficacy in targeting neural cells, enhances neuroprotective effects by delivering biomolecules that modulate inflammatory responses or promote cell survival, and facilitates the integration of GBMs with biological systems for better therapeutic outcomes.^17^

Various surface functionalization strategies exist, such as covalent and non-covalent functionalization, plasma hydrogenation, and nanoparticle functionalization, which will be discussed briefly below.^48^

#### 4.1.1 Covalent functionalization

Functionalized GO is the most intriguing and beneficial graphene derivative, although it is more expensive to produce for industrial applications, its cost is justifiable for biomedical uses.^17,64^ The covalent functionalization of GO results in chemical derivatives created through various routes similar to those used with other materials. The first method, click chemistry, involves efficiently joining small organic units under mild conditions, with the well-known azide–alkyne cycloaddition catalyzed by copper being a prime example. Other reactions fitting the click approach also exist. The second method, linker reactions, uses small functional molecules to act as bridges between the GO surface and other materials, essential when direct contact could cause denaturation, especially in biomolecules like proteins. The third method, direct chemical attachment, involves covalently bonding GO's oxygen functionalities to other molecules, with or without a catalyst, resulting in stable and reproducible immobilization.^48^ Both click chemistry and linker reactions are considered post-functionalization processes, whereas direct chemical attachment can be explained through the organic chemistry of GO's oxygen moieties. Carboxylic acids react through amidation or esterification, hydroxyl groups through silanization, silylation, or etherification, and epoxy groups through nucleophilic addition with amine-containing compounds. Miscellaneous reactions, often involving multiple oxygen moieties and functional groups, are common. Post-functionalization, unreacted oxygen groups on GO can be removed *via* a reduction reaction, partially restoring its graphenic character and producing covalently functionalized rGO with enhanced properties like conductivity and thermal stability, albeit with reduced hydrophilicity.^48,62^

#### 4.1.2 Non-covalent functionalization

Non-covalent functionalization of rGO provides a significant advantage by allowing for the immobilization of molecules on both sides of the graphenic basal plane without further chemical modification of the carbon lattice. This process helps prevent defects and preserves existing properties while introducing new ones. It involves π-stacking interactions, hydrophobic effects, van der Waals forces, electrostatic interactions, and hydrogen bonding, with material geometry sometimes playing a role.^48,60^ Non-covalent functionalization is often accomplished through simple mixing or incubation protocols for specific biomolecules and cells. While rGO is commonly utilized, GO can also be modified non-covalently, followed by chemical reduction. These modifications rely on electrostatic interactions and the pH of the medium. π-Interactions stabilize aromatic systems like dyes and polymers, whereas hydrophobic and van der Waals interactions are employed by non-aromatic molecules.^49,62^ Oxygen groups on the surface and edges facilitate the adsorption of polar or charged molecules. The combination of these interactions varies depending on the materials and conditions, which is crucial for the fabrication of new devices.^48^ A common strategy involves synthesizing systems through covalent and non-covalent interactions in multiple stages, leading to the creation of complex hybrid materials. While graphene functionalization spans various research fields, biological applications continue to be a rapidly growing area.^48,63^

### 4.2 Functionalization with biomolecules

GBMs are being increasingly studied for their potential in biotechnology and biomedicine, although this field is still developing. Graphenic surfaces are effective for interacting with various biomolecules, similar to CNTs. The integration of graphene with biomaterials leads to the creation of novel nanobiointerfaces through biofunctionalization, which involves attaching biomolecules—ranging from small organic groups to large proteins or cells—to the material. This process not only improves the biocompatibility and solubility of graphene but also enhances the immobilization and recognition of other molecules.^18^

Various biomolecules can be attached to graphenic materials through covalent and non-covalent interactions. For example, nucleic acids and aptamers link *via* π interactions due to their aromatic nucleobases. Phospholipid chains assemble onto GO and rGO layers through hydrophobic interactions, while proteins and enzymes can be immobilized through a combination of hydrophobic, π, and sometimes electrostatic forces, depending on the amino acid composition.^48^ Covalently functionalized graphene biosystems are primarily created through amidation or esterification reactions involving carboxyl groups, often facilitated by coupling reagents or through specific chemical reaction mechanisms.^63^ Additionally, functionalization may involve the opening of epoxy rings and modifications of hydroxyl groups. Proteins and polysaccharides stand out due to their complex chemical structures and abundance of functional groups.^48,62^

Amines are nucleophiles characterized by a basic nitrogen atom with a lone pair and can be categorized as primary, secondary, or tertiary. In chemical modifications of graphene, primary amines are most commonly used, either naturally occurring or introduced *via* amination.^48^ They react with carboxylic acids on graphene through condensation to form stable amide bonds. Additionally, amines can engage in nucleophilic substitution with epoxide groups, resulting in amine additions and ring openings. Chitosan is a notable macromolecule that can form amide bonds with GO. Amino acid side chains, particularly lysine, also participate in these reactions, alongside reactions involving alcohols, thiols, or carboxylate anions, which open up a wider range of interactions, particularly in proteins like keratin.^34,48,62^

## 5 Potential of GBMs on nervous system injuries and SCI

GBMs show a remarkable potential for neural tissue treatment due to their unique properties. Their different dimensional forms, including scaffolds, electrodes, nanostructures, injectable hydrogels, *etc.*, are able to repair neural injuries and SCI. The combination of GBMs with organic and inorganic materials, such as polymers or nanoparticles, can create multifunctional components that improve biocompatibility, mechanical strength, and electrical conductivity in nerve regeneration. In addition, these multifunctional composites can be used in smart or local drug delivery systems to enhance cell activity and therapeutic impacts.^2,65^ GO-based nanostructures and GQDs, by their high surface area and unique optical and electronic properties, are utilized as targeted drug or gene delivery carriers and imaging agents.^66,67^ These nanostructures can minimize secondary injury, improve delivery effects, and improve functional recovery. Graphenic scaffolds in 2D and 3D form can mimic the ECM and provide an appropriate substrate for axonal growth.^68,69^ Injectable hydrogels incorporated with graphene derivatives and biocompatible polymers can fill lesion cavities and form a network for cell infiltration, growth, differentiation into neurons, and regeneration. Furthermore, these hydrogels can be used as drugs/growth factor carriers to reduce inflammation and oxidative stress and to promote faster tissue restoration in SCI.^70,71^ Graphene-based biosensors are another substrate used for neural treatments. These devices can be utilized to monitor and record neural activity by detecting biochemical and electrical signals at the injured site.^65^Table 1 summarizes the applications and potential advantages of GBMs.

**Table 1:** Summary of GBMs applications and benefits for nervous system injuries and SCI

Application	Potential benefits	Ref.
Nerve regeneration	- Promote neurites regrowth and elongation	91
	- Provide stem cells differentiation into neural cells	
Nerve guidance conduits	- Provide a conductive space for nerve cell attachment and axonal growth	92
	- Promote the directional regeneration of damaged nerve fibers	
Neural interfacing devices	- Enable intimate integration with neural tissue for improved signal transduction	93
	- Enhance neuron-electrode coupling and recording/stimulation capabilities	
Electrical stimulation	- Highly conductive nature allows efficient electrical stimulation of nerves	94
	- Facilitate the regeneration and functional recovery of damaged nerves	
Piezoelectric stimulation	- Generate electrical signals in response to mechanical deformation	95
	- Provide a non-invasive method of nerve stimulation without external power source	
Scaffolds and matrices	- Highly customizable structure and properties to mimic the native extracellular matrix	72
	- Support the growth and differentiation of neural stem/progenitor cells	
Bioactive coatings	- Functionalization with growth factors, cell adhesion molecules, and other biomolecules	96
	- Enhance nerve cell proliferation, migration, and maturation	
Antioxidant properties	- Protect neural cells from oxidative stress-induced damage and apoptosis	97
	- Mitigate secondary injury mechanisms following traumatic or ischemic insults	
Anti-inflammatory effects	- Modulate the inflammatory response and reduce the formation of glial scar tissue	17
	- Create a more favorable microenvironment for nerve regeneration	

## 6 Cell therapy of nervous system injuries and SCI

Cell transplantation is a treatment option for SCI. Its goal is the replacement of damaged tissue with functional cells and regulation of the body's response to the injury. This can result in effects such as axon regeneration, immunomodulation, neuroprotection, neuronal plasticity, and remyelination. Several types of cells can be transplanted for SCI, each with its pros and cons.

Schwann cells, a type of glial cell that supports and insulates neurons, have been found to enhance neurite development and reduce inflammation following damage. Stem cells can promote neuroprotection and differentiate into neural cells (neurons and glial cells) under certain conditions, making them a versatile alternative for SCI treatment.^72^

Embryonic stem cells (ESCs), derived from blastocysts, are capable of differentiating into specific cell types prior to transplantation (such as neural precursor cells, neurons, or glia) due to their tumor generation potential. Some studies showed that ESC-derived cells can integrate into host tissue, avoid tumor formation, and improve motor function in animal models of SCI. However, the use of human embryos raises significant ethical concerns.^73,74^

Induced pluripotent stem cells (iPSCs) are created by reprogramming genes in mouse or human adult fibroblasts. They have been shown to promote axonal growth and neural network formation in animal models and a human clinical trial.^75^

Neural Stem Cells (NSCs) are pluripotent and self-renewing cells that isolated from the lateral ventricle, the hippocampus' dentate gyrus, and the spinal cord's central canal. They can differentiate into neurons, astrocytes, and oligodendrocytes. NSCs are capable of replacing injured cells and secreting neurotrophic compounds, reducing cell death, inflammation, and lesion size, inhibiting scar formation, and promoting functional recovery by establishing neural networks at injury locations.^76^

Mesenchymal Stem Cells (MSCs) are pluripotent, self-renewing cells with low immunogenicity and broad differentiation potential. MSCs can be produced from a variety of sources including bone marrow, adipose tissue, and the umbilical cord. They release growth factors and cytokines that promote neural regeneration. MSCs can protect neurons after SCI in two ways: (1) modulating the immune response and inflammation by suppressing inflammatory factors and changing macrophage polarization to the anti-inflammatory M2 phenotype and (2) producing neurotrophic factors such as BDNF, NT3, NGF, and GDNF. Recent research indicates that MSCs are capable of transferring mitochondria to neurons *via* gap junctions, hence lowering neuronal death. However, numerous challenges, such as immune response, tumorigenicity, and ethical concerns about hESCs, continue to drive interest in autologous and alternative cell therapies.^77–80^ The reliability and effectiveness of MSCs and NSCs for SCI therapy are investigated in clinical trials of NCT01676441 and NCT01772810, respectively.^81,82^

In this case, some important challenges must be solved. These include clarifying differentiation and regeneration mechanisms, optimizing cell production and transplantation protocols, and investigating the synergistic benefits of combining cell-based treatments with other therapeutic options before cell-based SCI therapies. More research is needed to discover the optimal type of cell, dosage, and timeline for transplantation in this situation.

### 6.1 Potential of GBMs on cell behavior of nervous system injuries and SCI

Interestingly, GBMs with different 2D and 3D morphology substrates, versatile surface properties, suitable biocompatibility, great physicochemical stability (for outgrowth and differentiation of stem cells on scaffolds), appropriate flexibility, and *in vivo* degradation ability have been investigated for increasing and differentiation of stem cells into particular lines (*e.g.*, osteogenesis, neurogenesis, oligodendrogenesis).^83–86^

In recent years, GBMs have been studied on the growth and differentiation of stem cells, mainly NSCs. Oxygen-containing functional groups (including carboxylic acid, carbonyl, hydroxyl, *etc.*) on the GO surface obtain hydrophilic features that are effective in cell attachment and growth.^87^

**Fig. 3:**
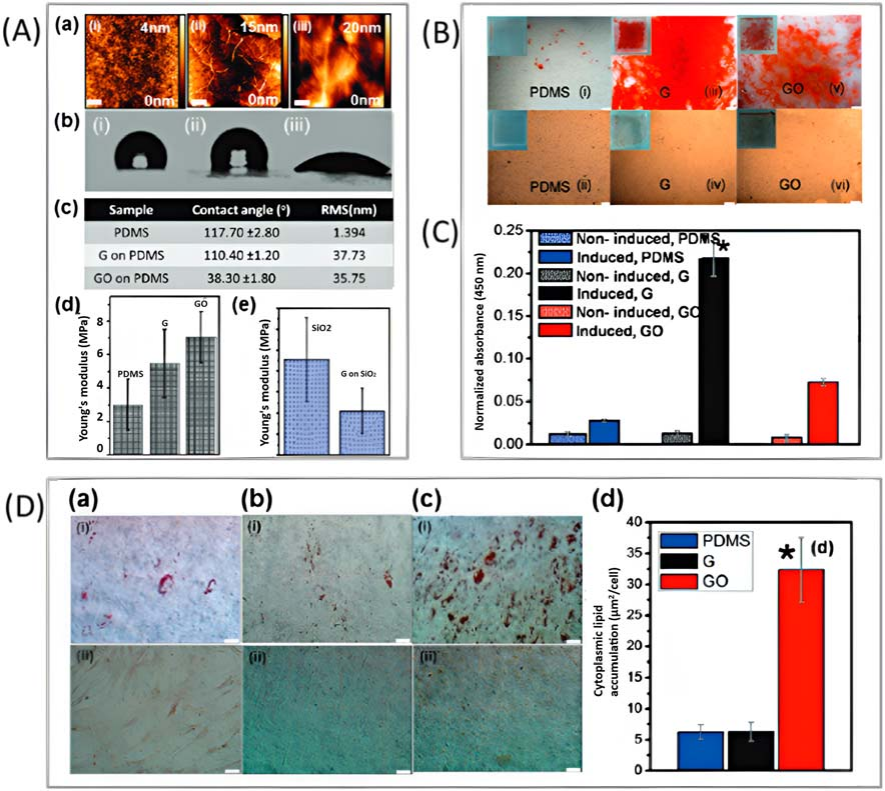
(A) Characterization of prepared substrates. (a) AFM, (b) contact angle and topography images, (c) table summarizing contact angle and roughness (rms), and (d) Young's modulus bar chart for (i) PDMS, (ii) graphene on PDMS, and (iii) GO on PDMS. (e) Young's modulus bar chart for SiO_2_ and graphene on SiO_2_. (B) Alizarin red staining of osteogenic differentiation after 12 days of incubation on PDMS, graphene, and GO with and without induction. Scale bars are 200 μm. (C) Quantification of Alizarin red staining in differentiated MSCs on graphene (*p < 0.05; *n* = 4 per group). (D) The extent of intracellular lipid formation was determined by oil red O staining after 14 days of induction on (a) PDMS, (b) graphene, and (c) GO with (i) and without (ii) induction. The scale bar is 50 μm. (d) The quantity of fat formation for MSCs differentiated on graphene, GO, and PDMS. *p < 0.05; *n* = 4 in each group. Reproduced with permission.^60^ Copyright *ACS Nano* 2011.

Additionally, these chemical groups play a crucial role in the proliferation and differentiation of various cells by providing different sticking properties of cells and proteins onto modified GBMs by amine, carboxylic acid, hydroxyl, and other specific functional groups. Prior research has demonstrated that GBMs can affect cell behavior due to the presence of aromatic rings in their structure and their capability to increase the local concentration of ECM such as fibronectin, collagen, and laminin through non-covalent binding. On the other hand, defaults in GO and rGO sheets can serve as active parts for cell attachment, proliferation, and differentiation.^63^ The first research by Lee *et al.*^87^ examined the effect of GO plates against CVD-grown graphene sheets on the proliferation and osteogenic differentiation of MSCs. They found that the higher osteogenic differentiation of MSCs on CVD-grown graphene (hydrophobic form) was more superior to GO (hydrophilic structure). This can be attributed to its function as an initial concentration site for osteogenic inducers. Furthermore, insulin, as a stimulator of fatty acid synthesis hormone, was found to adsorb onto CVD-grown graphene *via* powerful π–π interactions (Fig. 3).

Because of the strong properties of GO and its derivatives, researchers have extensively studied their effects on the proliferation and differentiation of various stem cells, such as human mesenchymal stem cells (hMSCs), iPSCs, and hNSCs. In the context of neural cells, GO and rGO sheets should be stimulated utilizing techniques like the flash photo, pulsed laser, and NIR irradiation to promote their differentiation into neurons.^88^

**Fig. 4:**
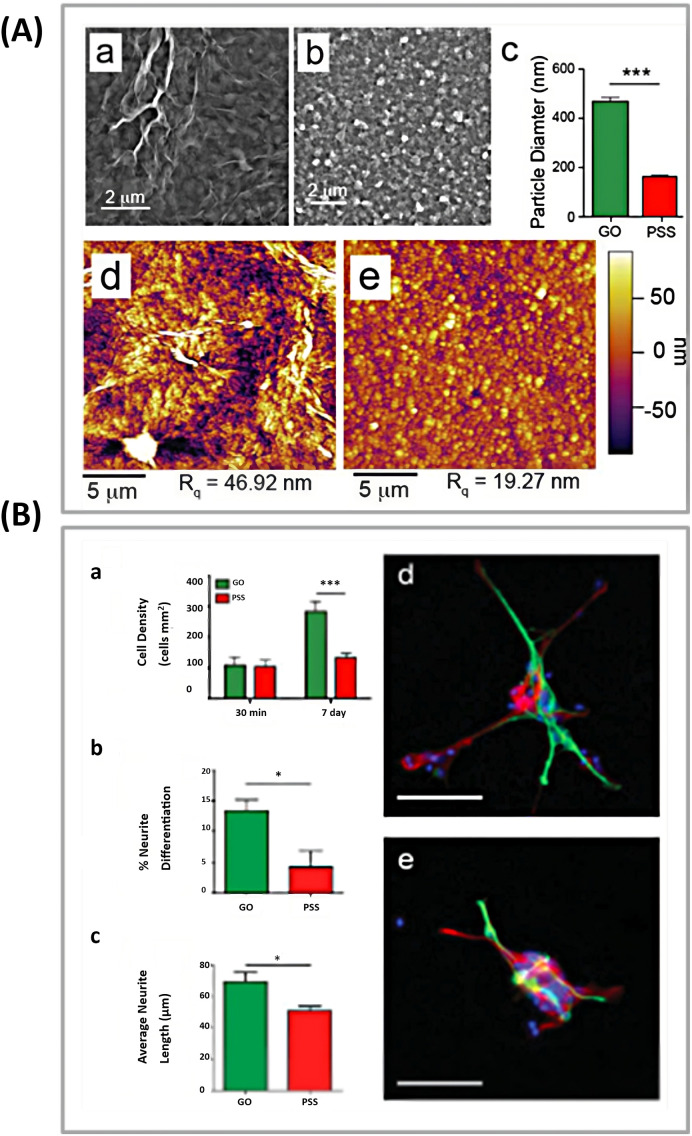
(A) Surface morphology of PEDOT films. SEM pictures of PEDOT treated with: (a) GO nanoplates or (b) PSS. (c) Determining the particle diameter in GO/PEDOT and PEDOT/PSS films. (***p < 0.001; *n* = 50). AFM pictures of PEDOT coated with (d) GO or (e) PSS reveal a higher root mean square (*R*_q_) surface roughness in GO/PEDOT nanocomposite films. (B) PEDOT substrates promote neural stem cell adhesion and differentiation. (a) Total cell counts on PEDOT films coated with GO or PSS after 30 minutes and 7 days in differentiation culture. (b) The proportion of neural differentiation, and (c) the average length of neurite development from differentiated neurons on PEDOT films at 7 days. The GO/PEDOT film leads to more differentiated neurons and longer average neurite length (*p < 0.05; *n* = 3). Typical fluorescence images of differentiated NSCs grown on (d) GO/PEDOT nanocomposite and (e) PEDOT/PSS substrate. The cells were labeled with neuron-specific β-III-tubulin (green), astrocyte-specific glial fibrillary acidic protein (red), and nuclei (blue). The scale bar in (d and e) indicates 50 μm. Reproduced with permission.^63^ Copyright from Wiley 2015.

Weaver *et al.*^89^ modified GO sheets with a conducting polymer poly(3,4-ethylenedioxythiophene) (PEDOT) and investigated its role in the differentiation of NSCs. They found that this biocompatible template could provide interferon-γ (IFNγ) and platelet-derived growth factor (PDGF) biomolecules as covalent cross-links, facilitating selective differentiation of NSCs. The template exposed to IFNγ and PDGF exhibited a higher number of neurons and oligodendrocytes, respectively (Fig. 4).

Besides the excellent capabilities of graphene and GO sheets (as 2D scaffolds) for cell differentiation and proliferation, 3D scaffolds with cell-arising micro-pores/channels, favorable topographies, and cell signal conductivity (both chemical and electrical signals) are highly required for the successful implementation of stem cells in a wide range of clinical applications. These applications relate to the creation and/or regrowth of body organs and the nervous system.^29^ To offer a quick summary, Table 2 summarizes the key findings on the bioapplications of graphene nanomaterials in nerve development and/or cell differentiation.

**Table 2:** Summary of the major findings of GBMs effects on neural growth and stem cell differentiation^a^

Material	Cell/stem cell	Differentiated cell	Main findings	Ref.
2D-graphene film	Mouse hippocampal cell	—	Increase germination of neurites and outgrowth of the cells	118
rGO membrane	MSCs	NSCs	Neural differentiation of MSCs through electrical stimulation route	91
Graphene films	Rat cortical primary neuron	—	Growth and proliferation of cells depended on surface chemistry rather than electrical conductivity	119
2D-graphene film	NSCs	Neuron	Formation of functioning neural networks, enhancing neural efficiency, and electrical signaling of the network	120
2D-GO and rGO powder	ADSCs	Neuron-like cells	GO was better for differentiation compared to rGO	121
2D-rGO nanogrids	hNSCs	Neurons & glia	Oriented and stretched-out differentiation of neurons	122
2D-laminin/GO-SiO_2_ nanoparticle hybrid	hNSCs	Neurons	Enhancement of the conjunction of neurons and axons, no alignment on MoS_2_ nanosheets	123
2D-graphene film	MSCs	Neurons	Promotion and guidance for the differentiation of stem cells	124
Ginseng-rGO films	hNSCs	Neurons & glial cells	Differentiation of hNSCs into neurons compared to glial cells	125
2D-GO-PCL nanofiber	NSCs	Oligodendrocytes	Growth of elongated cells on GO-PCL, preferential differentiation of NSCs into oligodendrocytes	126
rGO microfibers	NSCs	Neurons	Strength for adhesion and proliferation of NSCs compared to 2D graphene film	127
2D-carboxylic acid-functionalized GO	NSCs	Neurons or oligodendrocytes	Selective differentiation of NSCs into neurons or oligodendrocytes	89
2D-GO/Au	hADMSCs	Neurons	Increment of osteogenic differentiation	128
Silk fibroin-graphene film	iPSC	Neurons	Differentiation into neurons	129
3D-graphene foam	NSCs	Astrocytes & neurons	Excellent linkage between graphene foam and differentiated cells in the presence of the electrical field, 3D formation of neuronal networks	130
3D-bacterial cellulose- graphene foam	NSCs	Neurons	Differentiation of NSCs into neurons and development of neural networks	131
3D-porous GO	eNPCs	Neurons & glia	Selective growth of both neurons and glial cells	132
3D-rolled GO foam	hNSCs	Neurons & glia	Fabrication of neuronal fibers along the axis of the scaffold	133
3D-GO: Ce hydrogels	NSCs	Neuronal, astroglial and oligodendroglial lineage cells	Stiffer adhesion substrates promote differentiation to glial cell lineages, softer substrates enhance mature neuronal differentiation	134

a Abbreviations: MSCs (mesenchymal stem cells); ADSCs (adipose-derived stem cells); hNSCs (human neural stem cells); hADMSCs (human adipose-derived mesenchymal stem cells); iPSC (induced pluripotent stem cells); eNPCs (early neural progenitor cells).

## 7 Application of GBMs in the treatment of SCI (*in vitro* and *in vivo* studies)

Numerous GBMs have been the focus of extensive research due to their promising effects on spinal cord components, showing significant potential in both *in vitro* and *in vivo* models. López-Dolado *et al.*^90^ initially conducted an *in vivo* study on the response of rat spinal cord tissues to the flexible and spongy 3D-rGO scaffolds created utilizing the ice segregation-induced self-assembly (ISISA) technique. The *in vivo* specimen included a hemisection of around 8 mm^3^ at the right of the C6 level. In this study, rGO scaffolds were implanted at the injury site and covered with a gelatin layer. To investigate the subacute tissue replication, rats were sacrificed 10 days following surgery, with the collection of the spinal cord and other organs such as the liver, kidney, spleen, and lung tissues.

The results showed that rGO scaffolds organized a soft connection at the lesion site, with no significant diversity in the features of fibroglial scars compared to lesions without scaffolds. The scaffolds' porous design allowed cells to penetrate and move to the inner area. Immunohistochemical analysis proved positive for the β receptor of platelet-derived growth factor, which regulates blood vessel formation and prompts hematopoiesis, and vimentin, as an indicator of glial cells, connective tissue cells, and pericytes. Ultimately, neuronal cell quantities were protected in the perilesional areas, and no systemic harmful effects were observed. A further analysis was performed 30 days after surgery.

**Fig. 5:**
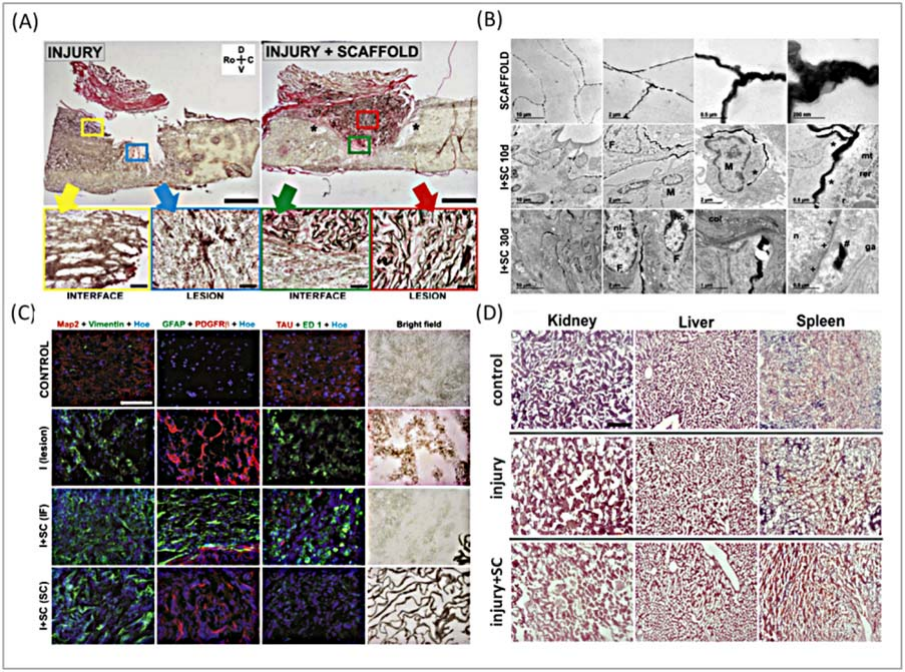
(A) Histological investigation of the interface and lesion places 30 days after damage using HvG staining. Scale bars measure 1 mm and 100 μm. Spinal cords are always orientated as shown by the following arrows: Ro – rostral, C – caudal, D – dorsal, and V – ventral. (B) Representative TEM micrographs of 3D rGO scaffolds following production and implantation in the spinal cord at 10 and 30 days. (C) Representative immunofluorescence pictures of the lesion site at 30 days after damage. Control samples are provided at the top for comparison. Bright field photos are supplied to validate the placement of the labeled cells relative to the scaffold. IF: interface; SC: scaffold. Scale bar: 100 μm. (D) Histological staining of the kidney, liver, and spleen 30 days after operation. Scale bar is 250 μm. Reproduced with permission.^81^ Copyright from *Biomaterials*, 2016.

As expected, cell and collagen penetration increased in the scaffold, along with a substantial decrease in vimentin-positive cells, growth, and the size and number of veins, all of which are crucial for the beginning of any regenerative procedure. All of these mutations can drive neuronal axon development into the scaffold, which was not observed at 10 days following operation surrounding functioning blood arteries (Fig. 5).

Markedly, no sign of inflammation, fibrosis, or atrophy was found in peripheral organs during the subacute phase. In the realm of *in vivo* studies, GO in the form of 2D nanosheets has been shown to produce concentration-dependent effects on the tail/spinal cord curve under hypoxic conditions in zebrafish embryos. This study emphasized the importance of environmental factors in influencing the biological interactions of nanomaterials.^98^ Additionally, a composite material made of graphene and collagen, created as 3D cryogels, was tested in rats. The composite demonstrated a capacity to boost axonal regeneration and encourage the polarization of M2 macrophages while also reducing neuroinflammation. This indicates that it offers both structural support and helps modulate the immune response.^99^ A composite of GO, chitosan, and polyethylene glycol (GO-CS-PEG) demonstrated significant improvements in functional recovery within the T10 segment of the mouse spinal cord by reducing inflammation, cystic cavity formation, and hemorrhage.^100^ In models of spinal cord hemisection using rats, the use of 3D porous scaffolds of rGO promoted tissue regeneration without forming fibrotic scars and enhanced M2-like macrophage infiltration. This was coupled with an increase in M2-like macrophage infiltration and angiogenesis, demonstrating the scaffold's ability to foster new blood vessel formation, which is vital for tissue repair and overall function.^68^ GO-PEG-diacerein hydrogels were effective in inhibiting astrocyte hyperactivation and inflammatory responses in rats, promoting axonal growth, and facilitating SCI repair. This highlights the therapeutic possibilities of GO composites in managing inflammation and supporting neural regeneration.^71^ Lastly, Mendonça *et al.*^101^ concentrated on the impacts of rGO and rGO functionalized with polyethylene glycol (rGO-PEG) on the BBB both *in vitro* and *in vivo*. Under *in vitro* studies, both rGO and rGO-PEG induced dose- and time-dependent toxicity. Especially, at a concentration of 100 μg mL^−1^, rGO constructed decreased toxicity in rat endothelial and astrocyte cells. For the *in vivo* study, rGO and rGO-PEG were given intravenously, and their adverse effects on the functionality of the BBB were assessed at various time points. Within 3 hours, both scaffolds showed significant decreases in astrocyte markers (GFAP and connexin-43, 47%), endothelial tightness (occluding), adherent junctions (β-catenin, 85%), and basal lamina (laminin, 134%). Interestingly, this impact vanished 7 days after the rGO administration, but the effects of the rGO-PEG composite remained steady and even grew with time. It can be attributed to the elevated concentration of intracellular reactive oxygen species and oxidative stress in rGO-PEG. These findings collectively underscore the immense potential of graphene-based nanomaterials in neural tissue engineering, though further research is essential to fully understand their interactions, optimize their applications, and ensure their safety in clinical settings. Table 3 summarizes outstanding studies on the performance of GBMs when placed in the spinal cord.

**Table 3:** Summary of investigation results of GBMs on spinal cord components

Material	Morphologies	Model	Research system	Major results	Ref.
GO	2D nanosheet	*In vivo*	Zebrafish embryo	Hypoxic condition beside the embryo, concentration-dependent tail/spinal cord curve	98
Graphene-collagen	3D cryogels	*In vivo*	Rat	Improves axonal regeneration and M2 macrophage polarization, reduces neuroinflammation	99
GO-CS-PEG	2D nanosheets	*In vivo*	T10 segment of mouse	Improve functional recovery, reduce inflammation, cystic cavity, and hemorrhage	100
GO-PEG-Au-CRL	2D nanosheets	*In vivo*	T10 segment of mouse	Nerve regeneration, cystic cavity, hemorrhage avoidance, and motor function improvement	112
rGO	3D porous scaffolds	*In vivo*	Hemisection of spinal cord at C6 in rats	Formation of flexible junctions with neural tissue, no addition of fibroglial scars, infiltration of scaffold into cells, affluence of vimentin^+^ and PDGFRβ^+^ cells, presence of M2-like macrophages	68
rGO	3D porous scaffolds	*In vivo*	Hemisection of spinal cord at C6 in rats	Collagen scaffold penetration increases, vimentin+ and ED1+ cells decrease, M2-like macrophages are augmented, angiogenesis occurs inside the scaffold, and new axons formation	90
Graphene-NH_2_ collagen	3D cryogels	*In vitro*–*in vivo*	Rat	Neuronal differentiation of BM-MSCs, regeneration of SCI by promoting cellular growth and migration, supporting neuro-inflammation	65
MoS2/GO/PVA	3D hydrogels	*In vitro*–*in vivo*	Mouse	Neural stem cells into neuron differentiation, recovery of locomotor function	155
GOPEG-diacerein	3D hydrogels	*In vitro*–*in vivo*	Rat	Inhibit astrocytes hyperactivation and inflammation reactions, axonal growth, promote the SCI repair	71
rGO-xanthan gum	3D porous scaffolds	*In vivo*	Rat	Growth of renewed nerve fibers, prevent the development of glial scar, and restore locomotor activity	108

## 8 Mechanism of GBMs in regeneration and stimulation of nervous system and SCI

Nervous system injuries have been prevalent during the last decade, caused by disorders or trauma that interrupt body function. SCI affects about 2.5 million people globally,^102^ while traumatic brain injuries occur in almost sixty-nine million (95% CI 64–74 million) people worldwide each year, most often in younger patients.^103^ These injuries frequently occur as a result of road traffic collisions, falls, or acts of violence. On all occasions, restoration of nervous tissue lesions and recovery of function remain highly demanding because of the complications of nervous system physiology and anatomy.

GBMs illustrate different significant features that make them promising candidates for the repair and regeneration of nervous system lesions. These materials have physical shape (planar geometry), surface topology, excellent mechanical stability for assistance outgrowth procedures, and flexibility to avoid subsequent damage to smooth surrounding tissues during movement. An additional remarkable feature of GBMs that has been shown to amplify nerve regeneration is their numerous electrical conductivity.^102,104^ GBMs, particularly scaffolds and hydrogels, cover the injured area while inhibiting astrocyte reaction and glial scar development in the lesion. They also bridge injury gaps and reconnect cells. These forms of GBMs resemble the ECM. GBMs' π–π interactions with amino acids on cell membranes promote cell adhesion, proliferation, differentiation, and axon regeneration.^105–107^ In one study, a xanthan gum-rGO gel scaffold was implanted into a spinal cord hemisection gap for six weeks. Immunofluorescent staining shows that nerve fiber growth is increased, but astrocyte activity and glial scar formation are reduced when compared to the control group. The scaffold's porous nature facilitated nerve fiber regeneration while limiting gelatinous scar formation. The authors believe the improved outcomes were due to the scaffold's electrical conductivity, which gave endogenous bioelectrical signals and boosted neural differentiation and cell adhesion for the guided growth of the regenerating nerves.^108^

GBMs, whether in nanoparticle, scaffold, or hydrogel type, serve as carriers.

**Fig. 6:**
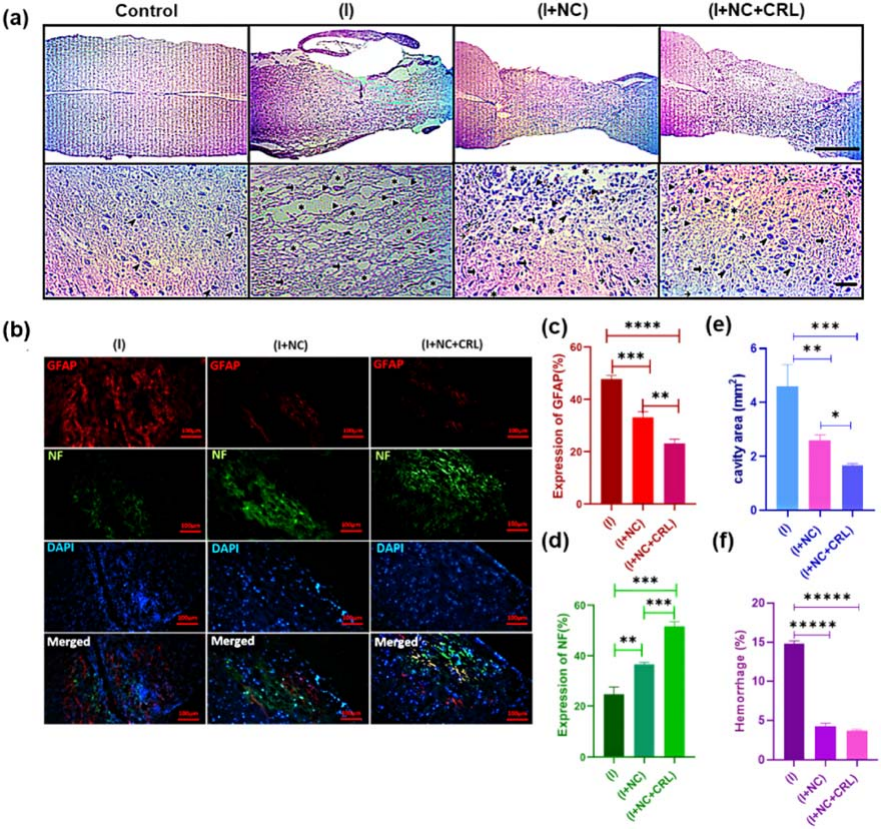
Shows H&E staining used to investigate the histopathology of neural tissue repair two weeks following surgery. Images from an optical microscope are shown at scales of 1 mm and 50 μm. (a) Magnified images of the groups showed cystic cavities (asterisks), hemorrhage (arrowheads), astrocytes (thick arrows), lymphocytes (thin arrows), and myelinated axons (thin arrowheads). (b) Immunofluorescence staining of sagittal slices of spinal cord tissue two weeks post-surgery. Scale bar is 100 μm. Quantitative data on NF staining (c), GFAP staining (d), cavity areas (e), and bleeding percentage (f) at longitudinal section lesion areas in the spinal cord. Mean ± SD, *n* = 12 per group. Adapted with permission.^112^ Copyright from *Nanoscale advances* 2024.

The specific functional groups on the surface of GBMs can interact with a broad variety of natural and synthetic molecules *via* noncovalent interactions (*e.g.*, π–π stacking, hydrogen bonding, electrostatic interactions) or covalent bonding. These advanced properties enable GBMs to hold, carry, or release proteins, drugs, genes, or growth factors at the injury site or cross neural physiological barriers such as the BBB and BSB to promote healing and neural regeneration.^109–111^ GBMs, especially GO derivatives and GQDs, can assist in reducing secondary damage produced by oxidative stress and inflammation. Recently, Yari-Ilkhchi *et al.*^112^ developed GO-PEG and GO-AuNPs nanocomposites to investigate their effects on mice with SCI, with and without local Cerebrolysin (CRL) administration at the lesion site. In particular, the *in vivo* performance of both nanocomposites resulted in neuroprotective and anti-inflammatory effects, including decreased cavity areas, hemorrhages, scar formation, and improved hind limb motor function 14 days post-injury. Local delivery of CRL displayed significant results in nerve regeneration in SCI (Fig. 6).

GBM-based electrodes or conductive substrates are very electrically conductive. These materials, when implanted in the body, can deliver electrical signals that sense and stimulate nerve activity. The electrical stimulation can proliferate cells, restore electrical connectivity in damaged areas, enhance neurite elongation, and improve motor and sensory function recovery.^113–115^ In addition, these electrodes can record neural impulses for controlling external equipment such as computers, wheelchairs, and robotic arms.^102,115^

**Fig. 7:**
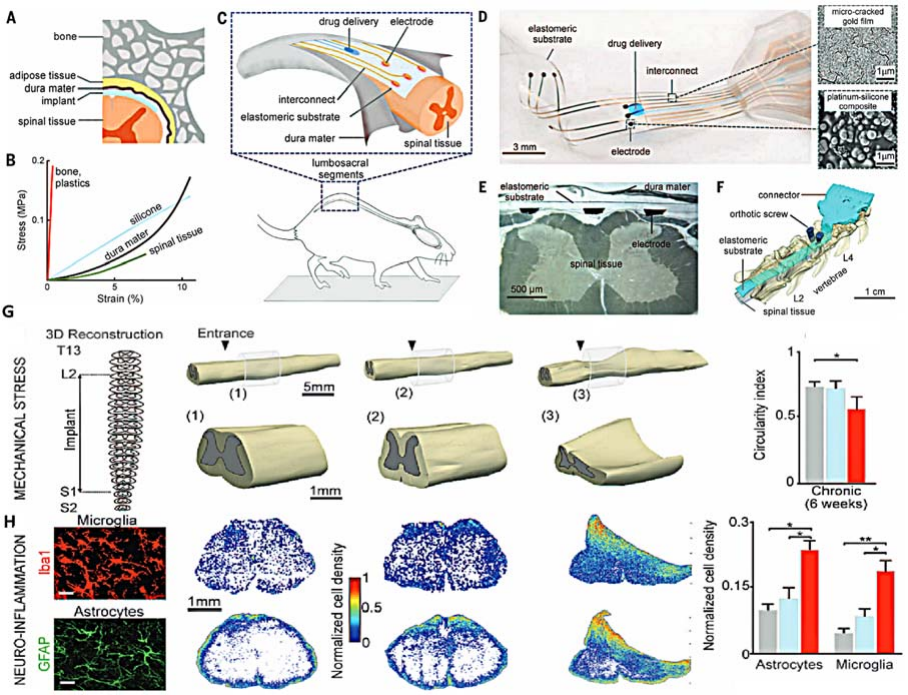
(A) A schematic cross section of the vertebral column with a flexible implant put into the spinal subdural region. (B) Strain–stress curves for spinal tissues, dura mater, and implant materials. (C) A representation of an e-dura implant placed into the spinal subdural area of rats. (D) Optical picture of an implant, with SEM images of the gold coating and platinum–silicone composite. (E) A 6-week e-dura insertion in the spinal subdural area is shown in the cross-section. (F) Restored 3D micro-computed tomography images of rats with e-dura injected into the spinal subdural distance, covering the L2 to S1 spinal segments. (G) 3D spinal cord reconstructions, containing improved views, six weeks following implantation. The arrowheads show where the implant enters the subdural cavity. Bar graphs show typical values of the spinal cord circularity index (4p × area/perimeter^2^). (H) Photographs of microglia (Iba1) and astrocytes (GFAP, glial fibrillary acidic protein) stained with neuroinflammation. Scale bars measure 30 mm. Reproduced with permission.^112^ Copyright from *Science* 2015.

Minev *et al.*^116^ fabricated a transparent and soft electrode with a shape and elasticity similar to those of dura mater to protect the membranes of the brain and spinal cord. The implanted electrode covered injured spinal segments L2 to S1 for 6 weeks to stimulate locomotor circuits without neuroinflammation. However, harder electrodes induced local spinal cord deformation, increased microglia and astrocyte presence, and impaired motor performance in just 1–2 weeks. Thus, the mechanical qualities of the electrode designs for SCI stimulation are crucial (Fig. 7). GO-polypyrrole (PPy) composite films on the Pt electrodes were synthesized and investigated for neurite functions by Deng *et al.*^117^

The negative charge of GO sheets and the positive charge of pyrrole cation radicals and PPy resulted in lower impedance by about 90% at 1 kHz and superior capacity compared to bare Pt microelectrodes and pure PPy-coated electrodes. The PPy/GO coatings created *via* the electrochemical co-deposition process appear promising for use in implantable neural probes.

## 9 Crucial aspects biodegradability, biocompatibility, and toxicity evaluation of GBMs

### 9.1 Biodegradability

After treatment, the cytotoxicity and degradation of the scaffolds are another significant concern that must be addressed. Graphene and its derivatives are increasingly being commercialized, making the degradation of graphitic materials after use crucial. GO is primarily prepared using aggressive oxidizing agents such as concentrated sulfuric acid, which can pose environmental hazards. Research indicates that certain microbial bacteria and enzymes are capable of oxidizing and degrading GBMs. The electron transfer in graphene compounds reduces their volume, facilitating their degradation process.^135^ This oxidation modifies the crystal structure, resulting in defects that enhance biodegradation. Myeloperoxidase from myeloid cells generates potent hypochlorous acid, which breaks down functional groups in graphene and aids its biodegradation.^136^ Factors such as the dispersion of GO and its surface charge play significant roles in this process. Additionally, lignin peroxidase, abundant in white rot fungi, has been shown to degrade graphene materials.^137^ New design techniques have been developed, incorporating molecules that bind to the surface of GO to increase its interaction with degradation-promoting enzymes. The biodegradability of GO largely depends on the functional groups present on its surface, with compounds like coumarin, catechol, and amino acids enhancing degradation.^138^ Common Gram-negative bacteria such as *E. coli* and *Shewanella putrefaciens* can rGO and facilitate extracellular electron transfer.^139^ While the health effects of graphene materials have been examined in various animal studies, the precise risks to humans are still under investigation. GO demonstrates remarkable stability in surface water, potentially impacting both biotic and abiotic elements of ecosystems. Therefore, it is essential to conduct comprehensive risk assessments and toxicity analyses before human exposure to these materials is permitted.^140^

### 9.2 Biocompatibility and toxicity of GBMs

Given the growing interest in using GBMs, the issue of biocompatibility and toxicity of them has gained significance. However, it's important to note that information on other carbon-based materials may not apply to GBMs due to differences in production methods, particle morphology, and surface chemistry. For example, unlike CNTs, the synthesis of GBMs typically does not involve metal catalysis, thus avoiding cytotoxicity and inflammation caused by residual metals.^141^ Biocompatibility is critical in evaluating GBMs, especially in their applications within biomedical fields. It refers to the ability of a material to carry out its intended function without causing any adverse reactions in biological systems. Research has demonstrated that certain graphene derivatives, such as GQDs and GO, show favorable biocompatibility profiles, as they are not absorbed into the bloodstream but eliminated through the stool. However, the increasing complexity of interactions between GBMs and biological systems requires a more thorough understanding of their biocompatibility.^142^ Graphene nanomaterials exhibit varying levels of toxicity across different organisms.^143^ In bacteria, they can be toxic to some species while remaining harmless to others, with toxic effects^144^ including antibacterial activity,^139^ oxidative stress,^145^ and cell membrane disruption,^146^ potentially influenced by iron (Fe^2+^) conjugation.^147^ In plants, the toxic effects of GBMs seem to be influenced by the concentration^148^ and duration of exposure, as well as the plant species used in the study, although cytotoxicity in plant cells is infrequently studied.^148,149^ In mammalian cells, the impact of graphene nanomaterials cannot be generally categorized as either cytotoxic or biocompatible, as it depends on factors such as concentration, functionalization, specific cell type, and the medium components, particularly fetal bovine serum (FBS), in which the cells are immersed. The toxic effects of GBMs can be mitigated through functionalization, which can also influence distribution within the body.^141^ The observed toxicity is related to the concentration and dosage of the materials, and when a tumor is present, the distribution tends to favor accumulation near tumor sites. Although the BBB is very selective, functionalized GBMs can permeate without interrupting its integrity, which is important to the development of new strategies for treating brain tumors and Alzheimer's disease.^143^

The toxicity and biocompatibility of GBMs have been evaluated through both theoretical analyses and studies using animal models. These investigations suggest that the toxicity of graphene is influenced by various physicochemical factors, including shape, size, oxidative state, functional groups, dispersion state, methods of synthesis, route, and dosage of administration, as well as exposure duration.^143^ Currently, there is a significant amount of data highlighting the toxicity of GBMs across different organs and systems in animals, making it challenging to include all findings in this review. Therefore, we have compiled a selection of literature and focused on specific *in vivo* and *in vitro* toxicological studies of GBMs, particularly concerning CNS.

#### 9.2.1 *In vitro* toxicity

Assessing *in vitro* cytotoxicity is a crucial preliminary step before the more expensive and elaborate *in vivo* studies. It identifies potential toxicity early on, guiding decisions for further research and optimizing resources.

##### 9.2.1.1 Dose, time, and morphology-dependent cytotoxicity

Zhang *et al.* explored the interactions of graphene (diameter 100–110 nm, thickness 3–5 nm) with rat pheochromocytoma PC12 cells using 3-(4,5-dimethylthiazol-2-yl)-2,5-diphenyltetrazolium bromide (MTT) and lactate dehydrogenase (LDH) assays, comparing the results with single-walled carbon nanotubes (SWCNTs). They observed over 70% cell death at 100 μg mL^−1^ of SWCNTs, whereas no cell death occurred at 0.01–10 μg mL^−1^ of graphene. About 15–20% cell death was seen at 100 μg mL^−1^ of graphene, attributed to agglomeration, ROS generation, and increased caspase-3 activation leading to apoptosis. This indicates a dose-dependent cytotoxicity trend, with graphene less toxic than SWCNTs.^150^ Vallabani *et al.* studied the toxicity of GO on normal human lung cells (BEAS-2B) after 24 and 48 hours of exposure at 10–100 μg mL^−1^ concentrations, finding a significant dose- and time-dependent decrease in cell viability and an increase in early and late apoptotic cells using the MTT assay.^151^

Yuan *et al.* assessed the cytotoxicity of GO on human hepatoma HepG2 cells using the MTT assay, DFDA fluorescence analysis, and 2D LC-MS proteome analysis. After 48 hours of exposure to 1 μg mL^−1^ GO, HepG2 cells showed 6% mitochondrial damage, an 8% increase in ROS generation, and no significant changes in apoptotic cell population, cell cycle, or expression of metabolic and cytoskeletal proteins. In contrast, cells treated with oxidized-SWCNTs (ox-SWCNTs) exhibited ∼20% mitochondrial damage, >100% ROS increase, ∼26% apoptotic cell population, and ∼30 differentially expressed proteins involved in metabolic pathways, redox regulation, cytoskeleton formation, and cell growth. These findings suggest GO is less cytotoxic than ox-SWCNTs.^152^ Additionally, Lv *et al.* reported that GO does not induce cytotoxic or apoptotic effects in human neuroblastoma SH-SY5Y cells at low concentrations (<80 μg mL^−1^), and it enhances retinoic acid-induced differentiation of SH-SY5Y cells, improving neurite length and MAP2 expression, indicating potential applications in neurodegenerative diseases.^153^

Talukdar *et al.* investigated the effects of graphene nanostructures—oxidized nanoribbons (GONRs), oxidized nanoplatelets (GONPs), and nano onions (GNOs)—on the toxicity and stem cell differentiation potential of hMSCs. hMSCs from bone marrow and adipose tissue were treated with various concentrations (5–300 μg mL^−1^) of GONRs, GONPs, and GNOs for 24 or 72 hours, and cytotoxicity was evaluated using Alamar blue and Calcein AM assays. The study revealed dose-dependent (not time-dependent) cytotoxicity, with concentrations above 50 μg mL^−1^ showing no cytotoxicity. TEM imaging indicated cellular and nuclear uptake of GNOs and GONPs. Additionally, all graphene nanostructures did not affect the adipogenic and osteogenic differentiation of hMSCs, suggesting graphene's potential use as labels for stem cell imaging and therapy.^154^

Chng *et al.* conducted a comparative study on the cytotoxicity of GONRs and GONPs. GONRs were derived from unzipping CNTs, whereas GONPs were synthesized from stacked graphene nanofibers.

Chng *et al.* conducted a comparative study on the cytotoxicity of GONRs and GONPs. GONRs were derived from unzipping CNTs, whereas GONPs were synthesized from stacked graphene nanofibers. *In vitro* cytotoxicity tests using MTT and WST-8 assays with human epithelial A549 cells indicated that GONRs exhibited significantly higher cytotoxicity across all concentrations (3–400 μg mL^−1^) compared to GONPs. This increased cytotoxicity was attributed to the higher amount of carbonyl groups (28.22% on GONRs *vs.* 11.06% on GONPs) and the high aspect ratio of GONRs (width × length: ∼310 × 5000 nm) *vs.* GONPs (∼100 × 100 nm).^156^

Akhavan *et al.* studied the cyto- and genotoxicity of rGONRs and rGO using hMSCs from umbilical cord blood. The fluorescein diacetate test showed significant cytotoxicity at 10 μg mL^−1^ of rGONRs within one hour, while the same level of cytotoxicity for rGO occurred at 100 μg mL^−1^ after 96 hours. The rGO's toxicity was linked to oxidative stress, whereas rGONRs caused DNA fragmentation and chromosomal aberrations, even at low concentrations (∼1 μg mL^−1^) due to cellular penetration. These findings suggest that graphene's cyto- and genotoxicity depend on the dose and shape of the nanomaterial (sheets *vs.* nanoribbons).^157^ Jaworski *et al.* studied the interactions of graphene platelets with human glioblastoma U87 and U118 cells. After 24 hours of incubation with 100 μg mL^−1^ graphene, they found 42% cell mortality in U87 cells and 52% in U118 cells. Interestingly, apoptosis was activated in U118 cells but not in U87 cells, where both apoptosis and necrosis were observed. These findings highlight the potential of graphene for anticancer therapy.^158^

##### 9.2.1.2 Toxicity of functionalized GBMs

Sasidharan *et al.* studied the cytotoxicity of pristine graphene and carboxylated GO (GO-COOH) using monkey renal cells at 10–300 μg mL^−1^ concentrations. Pristine graphene accumulated on the cell membrane, destabilizing F-actin alignment, whereas GO-COOH was internalized by cells and accumulated in the perinuclear region without membrane destabilization, even at 300 μg mL^−1^. These findings suggest that more oxidized, hydrophilic graphene nanoparticles may be more cytocompatible and effective for intracellular delivery.^159^ Matesanz *et al.* also found that poly(ethylene glycol amine)-functionalized GO sheets localized on F-actin filaments caused cell-cycle alterations, oxidative stress, and apoptosis in MC3T3-E1 murine pre-osteoblasts, Saos-2 osteoblasts, and RAW-264.7 macrophage cells.^160^

Yuan *et al.* investigated the cytotoxicity and distribution of NH_2_, COOH, and CO–N(CH_3_)_2_ functionalized GQDs in human neural glioma C6 and A549 lung carcinoma cells using MTT and Trypan blue assays. After 24 hours, no mortality, apoptosis, or necrosis occurred across all treatment concentrations (10–200 μg mL^−1^). Raman spectroscopy revealed intracellular accumulation of all three GQDs but no nuclear translocation.^161^

Horváth *et al.* evaluated the toxicity of GO and rGO in A549 human lung epithelial cells and RAW 264.7 mouse peritoneal macrophages using MTT, fluorometric DNA, and fluorometric microculture cytotoxicity assays. Cells treated with 0.0125–12.5 μg cm^−2^ of GO or rGO for 5 days showed dose-dependent cytotoxicity. Significant cell death was observed from day 2 in A549 cells and day 3 in RAW 264.7 macrophages at concentrations of 1.25–12.5 μg cm^−2^. Lower concentrations (0.0125–0.125 μg cm^−2^) of GO did not increase ROS production. GO internalized into phago endosomes without causing intracellular damage.^162^

Aggregation of pristine graphene in biological buffers can lead to greater cytotoxicity compared to GO derivatives, which disperse more readily. Das *et al.* reported higher cytotoxicity of GO sheets than rGO sheets due to the high density of oxidative functional groups on GO. HUVEC cells treated with GO and rGO (1, 5, or 10 μg mL^−1^) showed dose- and functionalization-dependent cytotoxicity. Smaller graphene nanosheets exhibited higher toxicity than larger ones due to increased intracellular interaction and uptake.^163^ However, Chong *et al.* found low cytotoxicity of PEG-dispersed GQDs for HeLa cells (up to 160 μg mL^−1^) and A549 cells (up to 320 μg mL^−1^).^61^

Teo *et al.* investigated the cytotoxicity of halogenated graphene sheets. They prepared chlorine-, bromine-, and iodine-doped graphene (TRGO-Cl, TRGO-Br, TRGO-I) from oxidized graphite and tested them on A549 cells at 0–200 μg mL^−1^ for 24 hours using MTT and WST-8 assays. Results showed dose-dependent cytotoxicity with TRGO-Cl having the highest (∼25.7% cell viability at 200 μg mL^−1^). The cytotoxicity levels followed the trend: TRGO-Cl > TRGO-Br > TRGO-I, dependent on halogen functionalization.^164^ In another study, Teo *et al.* reported the cytotoxicity of fluorinated graphene (FG) derivatives with varying fluorine content (1.5%, 42.6%, 50.7%). A549 cells treated with 0–400 μg mL^−1^ FG showed dose-dependent cytotoxicity, with higher fluorine content leading to greater toxicity.^165^ Similarly, Chng *et al.* evaluated highly hydrogenated graphene (HHG) in A548 cells, finding dose-dependent cytotoxicity at all concentrations (0–400 μg mL^−1^). The increased toxicity of HHG was attributed to the preferential adsorption of essential micronutrients on its hydrophobic surfaces compared to the hydrophilic surfaces of GO sheets.^166^

Sawosz *et al.* studied the cytotoxicity of arginine (Arg) and proline (Pro) functionalized rGO using U87 glioblastoma multiforme cells and tumors. Cells were treated with 50 μg mL^−1^ of rGO, rGO + Arg, and rGO + Pro for 24 hours. Results showed ∼40% cell death for rGO and ∼15% for rGO + Arg and rGO + Pro, compared to controls. Glioblastoma multiforme tumors in chicken embryos were injected with the compounds for 3 days, showing a greater reduction in tumor volume for rGO than for rGO + Arg and rGO + Pro. Histological analysis revealed necrosis and endothelial proliferation. rGO + Arg was found near microglial cells and blood vessels, while rGO + Pro was outside the cells in the tissue. Tumor cells need arginine for aggressive growth, placing rGO + Arg in active angiogenesis sites. Gene expression analysis indicated rGO + Arg downregulates MDM2 and increases NQO1 expression, suggesting anti-angiogenic and pro-apoptotic potential for glioblastoma multiforme therapy.^167^

##### 9.2.1.3 Size-dependent cytotoxicity

Akhavan *et al.* examined the cytotoxicity of rGONPs of varying sizes (11 ± 4 nm, 91 ± 37 nm, and 418 ± 56 nm) and as-prepared GO (3.8 ± 0.4 μm) using hMSCs. Cytotoxicity and cell viability were assessed with the FDA assay, ROS assay, RNA efflux, and Comet assay. Results revealed significant size-dependent cytotoxicity: 100 μg mL^−1^ of rGONPs (11 ± 4 nm) caused >95% cell death, decreasing with larger sizes. As-prepared GO showed the lowest cell death (∼20%). Additionally, rGONPs induced DNA fragmentation even at low concentrations (0.1 μg mL^−1^).^168^

Chang *et al.* studied the cytotoxicity of different sizes of GO on A549 lung adenocarcinoma cells. They used the CCK-8 assay to assess cell viability after 24–72 hours at GO concentrations of 10–200 μg mL^−1^. Small GO sheets (160 ± 90 nm) showed ∼67% cell viability, while larger sheets (430 ± 300 nm and 780 ± 410 nm) showed >80% viability. However, GO sheets of 780 ± 410 nm produced >50% higher ROS generation compared to smaller sheets. These results indicate that GO's cell viability and ROS generation depend on the size of the graphene sheets.^169^

Dasgupta *et al.* reported size-dependent cytotoxicity of GONRs after sonication, which reduces nanoparticle size. GONRs were dispersed in cell culture media using bath sonication (5 or 20 min) or probe sonication (1, 5, or 10 min), and MCF-7 and A549 cells were exposed to 20 μg mL^−1^ concentrations. LDH assay, Presto Blue assay, and ROS generation showed reduced metabolic stress in cells with probe-sonicated GONRs. No adverse effects were seen with non-sonicated and bath-sonicated GONRs. TEM analysis revealed smaller GONR fragments and carbonaceous debris post-probe sonication, possibly causing the observed cytotoxicity.^170^

Yue *et al.* reported that cellular internalization and response regulation depend on the lateral dimensions of GO. Six cell lines (PMØ, J774A.1, LLC, MCF-7, HepG2, and HUVEC) were exposed to GO sheets of different sizes (350 nm and 2 μm) at a concentration of 20 μg mL^−1^. After 48 hours, significant cytotoxicity (∼40–60% cell death) was observed, but cell viability was restored upon the removal of manganese (Mn). Cells treated with Mn-free GO at 20 μg mL^−1^ demonstrated ∼80–100% viability, underscoring the importance of purification during GO synthesis. PMØ and J774A.1 cell treated with 2–6 μg mL^−1^ of nano- and micro-sized GO exhibited similar intracellular accumulation. Uptake mechanisms indicated that 350 nm GO was internalized through filopodia wrapping, whereas 2 μm GO was internalized *via* direct penetration. Micron-sized GO induced stronger inflammatory responses and cytokine release, suggesting the size-dependent effects of GO sheets.^171^

#### 9.2.2 *In vivo* toxicology

A crucial step in evaluating the toxicity of graphene-based formulations involves assessing their dose- and time-dependent safety in small and large animal models through various administration routes, such as intravenous (IV), intraperitoneal (IP), and oral.

Singh *et al.* investigated the *in vivo* platelet aggregation of GO and rGO nanosheets. GO and rGO were intravenously injected into Swiss male mice at a dose of 250 μg kg^−1^ for 15 minutes, with collagen–epinephrine as the positive control and saline as the negative control. Histological analysis showed ∼48% thromboembolism for GO, ∼64% for collagen–epinephrine, and ∼8% for rGO, indicating GO's higher platelet activation. This may be due to the greater surface charge density of oxidized graphene. A follow-up study on amine-modified GO (NH_2_-GO) revealed no platelet aggregation or pulmonary thromboembolism, showing ∼46% blockage for GO and none for NH_2_-GO.^172^

Sasidharan *et al.* studied the long-term *in vivo* toxicology of pristine and functionalized few-layered graphene (FLG), FLG-COOH, and FLG-PEG in Swiss albino mice at 20 mg kg^−1^ for 1, 8, 30, and 90 days. Control mice received sterile saline. All mice survived 90 days, but FLG-treated mice had lower body weights on days 60–90. Using 99mTc labeling, FLG-COOH showed lung accumulation over 24 hours, while FLG-PEG was redistributed to the RES system (spleen and liver) after 12 hours. FLG-COOH caused thicker alveolar walls and spleen damage, whereas FLG-PEG caused minimal spleen injury. FLG and FLG-COOH induced liver degeneration and kidney necrosis, but FLG-PEG showed no necrosis. None of the treatments damaged the brain, heart, or testis, indicating graphene cannot pass the BBB.^173^

Zhang *et al.* studied the toxicity of dextran-functionalized GO (GO-Dex) in female Balb/c mice, IV injected at 20 mg kg^−1^ for 1, 3, and 7 days. H&E staining of liver sections showed increased black spots (GO aggregation) after 7 days, indicating clearance of GO-Dex from the liver. For biodistribution and pharmacokinetics, 125I-GO-Dex was injected at 4 mg kg^−1^, with blood collected at 4, 24, 72, and 168 hours. 125I-GO-Dex was initially found in multiple organs and later predominantly in the liver and spleen. Histological sections showed 125I-GO-Dex as black dots, decreasing over time, suggesting excretion *via* renal and fecal pathways. Small GO-Dex sheets were excreted really, while large sheets were excreted *via* the biliary pathway.^174^

Zhang *et al.* studied GO distribution and biocompatibility in male Sprague Dawley rats, IV administered at 1 and 10 mg kg^−1^ doses. Histopathological analysis 14 days post-injection showed no changes in the lung, liver, spleen, and kidneys for the 1 mg kg^−1^ dose. However, the 10 mg kg^−1^ dose caused pulmonary edema, granulomatous lesions, inflammatory cell infiltration, and lung fibrosis due to high accumulation and slow clearance. Biodistribution tracking with 188Re-labeled GO revealed GO clearance from blood and accumulation in the lungs, liver, and spleen, being taken up by mononuclear phagocytes in the reticuloendothelial system. These findings suggest GO is biocompatible but may pose safety concerns at higher concentrations due to lung accumulation.^175^

Wang *et al.* studied the biocompatibility of GO in female Kunming mice *via* tail vein injections at doses of 0, 0.1 mg (low), 0.25 mg (medium), and 0.4 mg (high). No toxicity was observed at low and medium doses. However, 4 out of 9 mice given the high dose died after one week due to airway blockage from GO accumulation. Histology after 1, 7, and 30 days showed long-term accumulation of graphene in the liver, kidney, and spleen. Granuloma formation, neutrophils, and foamy alveolar macrophages were seen in the lungs, indicating a foreign body immune response. No brain accumulation was observed, suggesting GO cannot cross the BBB. These results suggest GO is non-toxic at low concentrations but causes irreversible airway damage and chronic pulmonary toxicity at high concentrations.^176^

Liu *et al.* reported dose- and size-dependent toxicity and biodistribution of GO sheets. Male ICR mice were IV injected with small and large GO sheets (s-GO and l-GO) labeled with 125I, tracking tissue biodistribution and organ accumulation for 2–180 minutes post-injection at 1–10 mg kg^−1^ doses. s-GO mainly accumulated in the liver, with some in the lungs and spleen, but cleared to ∼11% in the liver and <1% in the lungs after 180 minutes. Conversely, l-GO showed higher lung accumulation (∼19% after 180 minutes). TEM analysis revealed intracellular s-GO in phagocytic cells and l-GO in lung cell gaps. GO's size-regulated biodistribution was due to different nanoparticle aggregation states. Less dispersed GO formed larger GO-protein complexes filtered by pulmonary blood vessels, with s-GO aggregating into large particulates at higher doses. s-GO had a blood half-life of 2.2 minutes (T1/2 alpha) and 170 minutes (T1/2 beta), while l-GO had 1.8 minutes (T1/2 alpha) and 102 minutes (T1/2 beta), suggesting longer blood retention for s-GO.^177^

Yang *et al.* reported the *in vivo* biodistribution and photothermal activity of PEG-functionalized nano graphene sheets (NGS-PEG). Cy7 dye-labeled NGS-PEG was intravenously injected into tumor-bearing Balb/c mice at 20 mg kg^−1^, and organs were harvested at 1, 6, and 24 hours. NGS-PEG showed significant tumor accumulation due to leaky vasculature and low accumulation in RES organs. After 24 hours, strong fluorescence in the kidneys indicated renal excretion of small NGS particles. NGS-PEG showed no toxicity, with no deaths or significant weight loss. Tumors exposed to an 808 nm laser post-NGS-PEG administration disappeared within a day, leaving scars that healed in a week with no regrowth after 40 days. These results suggest that PEG-functionalized graphene is suitable for *in vivo* photothermal therapy.^178^ In another study, Yang *et al.* reported the long-term biodistribution and pharmacokinetics of 125I-labeled NGS-PEG in Balb/c mice at 4 mg kg^−1^. Blood samples were taken over 0–25 hours, and organs were harvested up to 60 days post-injection. NGS-PEG initially accumulated in several organs, later concentrating in the liver and spleen. H&E staining showed decreasing NGS-PEG aggregates, indicating removal from the RES system. Smaller NGS-PEG (10 nm) was cleared renally, while larger aggregates were excreted *via* the biliary pathway. Blood biochemistry and hematology analyses showed normal levels, suggesting no toxic effects to the liver and kidneys. These results indicate that NGS-PEG does not exhibit long-term *in vivo* toxicity in mice.^179^

Kanakia *et al.* reported subacute toxicity of dextran-functionalized graphene nanoplatelets (GNP-Dex) in Wistar rats, given intravenous doses of 1, 50, and 100 mg kg^−1^ three times a week for three weeks. No toxicity was observed at 1 mg kg^−1^ and 50 mg kg^−1^ doses, with normal body weight, blood pressure, breathing, and heart rate. However, at 100 mg kg^−1^, 2 out of 8 rats died after 2 weeks. Blood analysis showed normal kidney function, though ALT and ALP levels were elevated. Histology after 3 weeks revealed GNP-Dex in hepatic Kupffer cells and pulmonary alveolar macrophages, increasing with dose. No adverse effects were seen in the brain, heart, spleen, and kidney.^180^

Mullick Chowdhury *et al.* studied the *in vivo* vasoactivity of GNP-Dex using a hamster cheek pouch model. GNP-Dex was administered at 1–50 mg mL^−1^ to the excised left cheek pouch tissue of hamsters. No significant effect on arteriole diameters was observed at 0.1, 0.5, 10, and 50 mg mL^−1^ doses. However, 35 mg mL^−1^ of FDA-approved dextran caused ∼23% dilation of arcade arterioles and ∼63% dilation of terminal arterioles. The lack of dilation with GNP-Dex and increased dilation with dextran suggests the minor vasoactive effects of GNP-Dex are due to the dextran coating on GNPs.^181^

Kanakia *et al.* evaluated the histopathology and biodistribution of GNP-Dex in male Wistar rats, administered intravenously at doses of 1–500 mg kg^−1^ for 1 and 30 days. The maximum tolerable dose (MTD) of GNP-Dex is between 50 and 125 mg kg^−1^, with a blood half-life of ∼30 minutes. GNP-Dex accumulated most in the liver and kidney after day 1, reducing 2–4-fold after 30 days, indicating clearance *via* the RES system. At 50 mg kg^−1^, GNP-Dex showed higher blood concentration than at 500 mg kg^−1^ after 30 minutes. Most GNP-Dex nanoparticles were excreted *via* feces (60–90%) within 24 hours, with small amounts in urine. High treatment concentrations (250 μg mL^−1^) caused histopathological changes in the heart, lung, liver, kidney, and spleen, but no adverse effects were observed in the brain. Hematological and cardiovascular parameters remained normal up to 125 mg kg^−1^, suggesting GNP-Dex is non-toxic with an MTD of 125 mg kg^−1^.^182^

Jasim *et al.* studied the *in vivo* biodistribution of chemically functionalized graphene (GO-DOTA) labeled with ^111^In in C57BL/6 mice at 200 μl dosage. After 1, 2, and 24 hours post-injection, ^111^In-DOTA-GO accumulated in the bladder and was excreted *via* urine, with no fecal elimination. Maximum accumulation was in the liver and spleen, with later translocation from the liver to spleen. No organ damage was observed, indicating that chemically functionalized GO sheets are non-toxic and have distinct biodistribution and excretion behaviors compared to pristine or non-covalently functionalized graphene sheets.^183^

In oral administration, substances are taken through the mouth for systemic effects. Fu *et al.* investigated the development of mice offspring after maternal mice were given GO at 0.5 and 0.05 mg mL^−1^ in drinking water from 1 to 38 postnatal days (PND). Filial mice received GO during the suckling period (1–21 PND) and normal water during the weaning period (22–38 PND). After 21 and 38 days, significant decreases in body weight, body length, and tail length were observed in the 0.5 mg mL^−1^ group compared to controls. Blood analysis showed no significant differences in ALT, AST, BUN, and CREA levels for both GO groups. Pathological examination of mice given 0.5 mg mL^−1^ GO showed severe atrophy in the heart, lung, spleen, kidney, and liver. H&E staining revealed increased villi length and duodenum width in the small intestine. These results indicate that GO can negatively affect the development of filial mice during lactation.^184^

Zhang *et al.* studied the short- and long-term effects of rGO on locomotor activity, neuromuscular coordination, balance, anxiety, learning, and memory in male C57b/6 mice. Mice were given 60 mg kg^−1^ of HEPES buffer-dispersed rGO *via* oral gavage every 24 hours for 5 days. rGO-treated mice maintained normal body weight, organ weight, and instinctive behaviors compared to controls. However, during the initial 3–4 days, mice showed decreased neuromuscular coordination and locomotor activity. These parameters returned to normal by 15- and 60 days post-treatment. Blood biochemistry, liver and kidney function, and neuron morphology in the hippocampus remained normal. rGO exposure resulted in a short-term decrease in neuromuscular coordination and locomotor activity, which returned to normalcy after a few days without affecting learning, memory, anxiety, or exploratory behaviors.^185^

Wu *et al.* investigated the toxicity of GO on the nematode Caenorhabditis elegans at doses ranging from 0.1 to 100 mg L^−1^, administered orally. Both acute (24 hours) and prolonged exposure (from larva to adult) to GO mixed with nematode food was examined for lethality, growth, reproduction, and locomotion. Prolonged exposure at concentrations of 0.5 mg L^−1^ and higher resulted in significant damage to primary (intestine) and secondary (neurons and reproductive) organs. GO also induced villi loss and translocation into the intestinal walls, increased defecation cycles, and caused a hyper-permeable intestinal barrier. These findings suggest that GO exposure in the environment could have long-term adverse effects on nematodes, worms, and other environmental organisms.^186^

Yang *et al.* reported the *in vivo* toxicity of PEG-functionalized GO administered IP and orally in female Balb/c mice. PEG-functionalized and 125I-labeled nano GO (nGO-PEG), rGO-PEG, and (nrGO-PEG), with diameters of 25, 50, and 27 nm, respectively, were administered intraperitoneally at 50 mg kg^−1^ and orally at 100 mg kg^−1^. Animals were euthanized at 1, 7, 30, and 90 days post-IP administration and 1, 7, and 30 days post-oral injections. Major organs were collected for histology and biodistribution analysis, and blood was collected for a complete blood panel and serum biochemistry. The radioactivity of GO formulations was undetectable one week after oral administration, indicating negligible uptake. However, after IP administration, PEGylated GO showed high accumulation in RES organs (liver and spleen) at 1 and 7 days. Larger rGO-PEG demonstrated higher uptake than smaller nGO-PEG and nrGO-PEG formulations. No animal deaths, weight loss, inflammation, or significant changes in blood or serum biochemistry were observed after 90 days post-IP administration, suggesting no toxicity. The biodistribution and clearance of PEGylated GO depend on size, surface coating, and administration route.^187^

Ali-Boucetta *et al.* investigated the *in vivo* pathogenicity of highly pure, colloidally stable GO dispersions. Conventional GO (cGO, >0.10 μm^2^) was purified to obtain highly pure GO (pGO, 0.01–0.02 μm^2^), both with similar chemical groups. pGO was administered IP at 50 μg per animal for 1 and 7 days, with CNTs as positive controls. After 1 day, pGO showed no change in polymorphonuclear leucocyte (PMN) or protein levels, while CNTs increased PMN count 2-fold. After 7 days, CNTs caused macrophage and giant cell accumulation with collagen deposition, but pGO did not. These results indicate that highly pure GO sheets show no inflammation or granuloma formation up to 50 μg per animal intraperitoneally.^188^

Sahu *et al.* investigated the biocompatibility of GO in pluronic gels implanted IP in Balb/c mice. Mild inflammation was noted at 3 weeks, but no chronic inflammation, necrosis, or hemorrhaging was observed after 8 weeks. No gel degradation was detected.^189^

Strojny *et al.* studied IP toxicity of GO, graphite, and nanodiamonds in Wistar rats. Injected nanoparticles showed aggregation near the injection site and some accumulation in the liver. No adverse health effects were observed, and blood and liver enzyme levels remained normal, indicating good liver biocompatibility.^190^

#### 9.2.3 Toxicity and biocompatibility of GBMs in the CNS and SCI

Graphene's advances in neurosurgery are notable, particularly in drug and gene delivery for brain tumor treatment, biocompatible devices, biosensing, and bioimaging. While these applications are promising, emerging studies have raised concerns about graphene's effects on brain tissue. In chicken embryos, pristine graphene flakes reduced RNA levels and DNA synthesis, adversely impacting brain development and causing a typical brain ultrastructure. This highlights the need to balance the potential benefits and risks of graphene in medical applications.

In the context of SCI treatments, interfacing GBMs with intracellular organelles remains inconclusive. Progress requires understanding both the positive and negative effects of these nanomaterials on cellular machinery. However, comparing studies is challenging due to varying protocols, cell lineages, GBM concentrations, time points, and fabrication methods. Based on other studies, conflicting results on GBM nanotoxicology arise from variations in physicochemical properties like size, layer count, oxidation state, and shape, leading to different cell membrane interactions that may cause inflammation, oxidative stress, genotoxicity, and cell death. Herein, the focus is on understanding how stem cells and CNS neural cells respond to GBM nanosheets to tackle SCI challenges effectively.^191^

##### 9.2.3.1 Neurons specific interactions with GBMs

Currently, our understanding of the cytotoxic, molecular, and physiological effects of exposing neurons to GBMs is still limited.^3^ Ramini *et al.* recently investigated how graphene and GO nanosheets interact with cultured primary neurons, revealing several key findings. Many nanosheets failed to enter the cells due to aggregation. The nanosheets that did internalize followed the endolysosomal pathway without impacting neuron viability or morphology. Notably, long-term exposure to GO (2 weeks) significantly influenced neuronal activity by favoring inhibitory synapses over excitatory neurotransmissions. These specific interactions with neurons emphasize the need for further research to fully comprehend the effects of GBMs on neural cells.^192^ In another study, Rauti *et al.* confirmed that, unlike cytotoxic large GO flakes (10–15 μm), smaller GO nanosheets (50–500 nm) were biocompatible. They effectively interacted with presynaptic glutamatergic terminals of neurons, leading to the downregulation of excitatory synaptic activity.^193^ The same group conducted an *in vivo* study on rats, showing that intrahippocampal delivery of GO nanosheets initially triggered glutamate release, enhancing excitatory synaptic activity. This was followed by an obstruction of the presynaptic exocytosis process, significantly limiting glutamatergic neurotransmission. Inhibitory (GABAergic) synapses remained unaffected, and the nanomaterial nearly disappeared from the injection site after 72 hours, preventing aggressive microglial activation.^194^ Intraspinal administration of GO nanosheets replicated similar results, decreasing excitatory synapses in the spinal neural.^195^ Network and reducing locomotor activity in zebrafish embryos GO's performance as a biocompatible inhibitor of glutamatergic neurotransmission supports its potential for neuroprotective approaches targeting SCI, particularly for reducing glutamate-induced excitotoxicity during secondary injury. However, the ability of GO to produce CNS toxicity remains controversial. An *in vivo* study showed that GO could translocate from the aqueous environment to the brain of zebrafish larvae, inducing Parkinson's disease-like symptoms, such as disrupted locomotor activity and loss of dopaminergic neurons.^196^ Unlike GO, rGO nanosheets in the zebrafish spinal cord increased locomotor performance by boosting local synaptic activity. This impact on swimming performance was delayed but long-lasting, noticeable from 4 hours of exposure and persisting after 24 hours. GO flakes had earlier (after 2 hours) but temporary inhibitory effects, disappearing after 24 hours. In rat postnatal hippocampal cultures, rGO nanosheets enhanced neuronal network excitability by increasing the amplitude and frequency of spontaneous postsynaptic currents and the number of presynaptic vesicles containing glutamate.^197^ This finding contrasts with a study by Kang *et al.*, which indicated that neuronal cells (PC12 and embryonic cortical neurons) can oxidize rGO nanosheets *via* intracellular ROS *in vitro*. The higher oxygen content in these nanomaterials disrupted actin filament dynamics, reducing neurotransmission, as shown by the lower amplitude and frequency of excitatory spontaneous postsynaptic currents. The discrepancies between the studies may stem from differences in cell types (postnatal hippocampal *vs.* PC12/embryonic cortical neurons), physicochemical properties of rGO nanosheets (total oxygen: 6.1% *vs.* 11.4%; size: 300 nm to a few μm *vs.* 98.4 ± 21.3 nm), and treatment conditions (rGO concentration: 10 μg mL^−1^*vs.* 20 μg mL^−1^; exposure time: 6–8 days *vs.* up to 12 hours).^198^ Preliminary nanotoxicological profiles of rGO indicate that the route of administration is crucial for determining biocompatibility. Rats showed no CNS toxicity after intravenous injection.^199^ However, a short-term decline in locomotor activity and neuromuscular coordination was observed when rGO nanosheets were administered orally.^185^ In addition to modulating neuronal cells, the delivery of GQDs into injured spinal cords shows significant reparative potential. Tosic *et al.* used an autoimmune encephalomyelitis rat model to evaluate GQDs in counteracting neuroinflammation. Analysis of spinal cord tissue revealed that GQD internalization in immune and CNS cells boosted the MAPK/Akt signaling pathway, inducing substantial neuroprotective effects on neurons and oligodendrocytes.^200^ Kim *et al.* demonstrated that GQDs can penetrate the BBB without inducing toxicity, ameliorating critical Parkinson's disease outcomes such as neuronal death, low synaptic protein levels, and mitochondrial dysfunction. Further research in this area is highly encouraged.^201^

##### 9.2.3.2 Glial cells' specific interactions with GBMs

Glial cells play a crucial role in regulating and supporting neural circuit function, making the re-establishment of local cell–cell interactions essential for the success of SCI therapies. GBMs could emerge as promising agents to enhance neuroglia responses at molecular, structural, and functional levels.^202^ Chiacchiaretta *et al.* demonstrated that graphene and GO internalization didn't affect astrocyte viability *in vitro*, as both followed an endolysosomal pathway. However, astrocytes altered their cytoskeleton, shifting from a standard epithelioid morphology to an asymmetric shape with long processes, indicating a more differentiated state. Astrocytes interfacing with GO nanosheets regulated the extracellular environment and neuronal activity by upregulating K^+^ buffering and glutamate uptake, which accelerated the maturation of cocultured neurons and increased inhibitory synapse density.^203^ Bramini *et al.* explored the molecular modulation triggered by GBMs in astrocytes, linking the chemical compositions of graphene and GO nanosheets to distinct proteomic and lipidomic profiles.^204^ A recent study showed that GO nanoflakes increased astrocytes' production and release of microvesicles involved in intercellular communication *in vitro*. Isolating and delivering these GO-derived microvesicles into a cortical primary culture enhanced synaptic activity and softened neuronal mechanical properties linked to vesicular fusion in the plasma membrane.^205^ Few studies address CNS defensive mechanisms against GBMs. A recent report showed that, *in vitro*, rGO nanosheets reduced ROS and proinflammatory cytokine release compared to GO nanosheets after 24 hours. Only macrophages exposed to high GO concentrations (10 μg mL^−1^) significantly expressed CD80, a pro-inflammatory marker.^206^ In a 3D mouse spinal cord culture, long-term (2 weeks) and high-concentration (25 and 50 μg mL^−1^) GO nanosheet exposure led to significant microglia proliferation without a pronounced release of pro-inflammatory molecules or astroglial response.^207^ Despite maintaining neuronal viability, both excitatory and inhibitory synapses were downregulated, unlike hippocampal neurons, which only limited glutamatergic neurotransmission *in vitro* and *in vivo*.^194^

#### 9.2.4 Toxicity mechanisms of GBMs

**Fig. 8:**
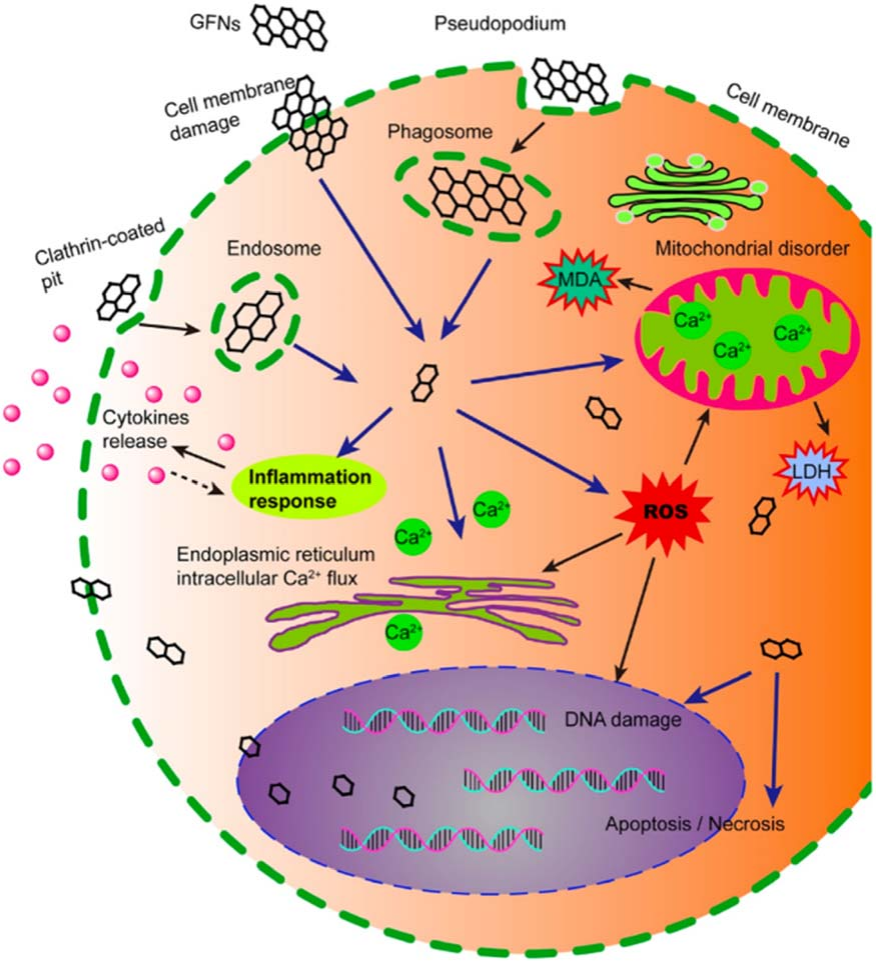
The schematic diagram showed the possible mechanisms of GFN cytotoxicity. GFNs get into cells through different ways, which induce ROS generation, LDH, and MDA increase, and Ca^2+^ release. Subsequently, GFNs cause kinds of cell injury, for instance, cell membrane damage, inflammation, DNA damage, mitochondrial disorders, apoptosis-sis or necrosis. Reproduced by CC-BY license.^192^ Copyright from Springer Nature.

While the physicochemical properties and toxicity of GBMs have been investigated, the fundamental mechanisms driving their toxicity are still not fully understood. Three different mechanisms have been suggested and confirmed for graphene toxicity in cell culture and the body: (i) direct interaction between graphene edges and cell damage (cell nuclei and membrane); (ii) formation of ROS; and (iii) blockage of cells *via* aggregation of graphene sheets on the cell surface.^29,191^ The proposed mechanisms of GBM toxicity are illustrated in Fig. 8.

##### 9.2.4.1 Physical destruction

GBMs in the nanoscale is unique compared to other nanoparticles due to their two-dimensional structure with sp^2^ carbons. This physical interaction is a source of cell membrane toxicity.^208^ GBMs can bind with α-helical peptides due to their surface curvature. When the concentration of pristine graphene exceeds 75 μg mL^−1^, it adheres to RAW 264.7 cell surfaces, abnormally elongating cell membranes.^209^ Strong hydrophobic interactions between GBMs and the cell membrane stretch F-actin filopodia and cytoskeletal cells. The sharp edges of GBMs can cut or insert into cell membranes, directly destroying bacterial cell membranes and releasing intracellular components.^144,210^

##### 9.2.4.2 ROS production

Elevated levels of ROS in cells arise due to oxidative stress, which increases the activity of antioxidant enzymes such as superoxide dismutase (SOD), glutathione peroxidase (GSH-PX), and catalase. ROS production leads to macromolecular cell damage, lipid membrane breakdown, protein denaturation, DNA fragmentation, and mitochondrial dysfunction, which affect cell signaling and metabolic pathways.^61,211,212^ GO interaction with cells increases ROS generation, contributing to aging, carcinogenesis, and mutagenesis. Dose-dependent administration of GO decreases SOD and GSH-PX enzyme activity. Exposure of HLF cells to GO damages DNA and leads to apoptosis. Pristine graphene and proapoptotic proteins like Bcl-2 (Bim and Bax) activate ROS generation, altering MAPK and TGF-β signaling pathways, activating effector proteins and caspases, and inducing apoptosis. Signaling pathways like MAPK, TGF-β, and TNF-α induce inflammation, tissue necrosis, and apoptosis.^213,214^

##### 9.2.4.3 Mitochondrial damage

Mitochondria, the cell's energy production centers, play a crucial role in signaling pathways and apoptosis regulation. Exposure to GO and carboxyl of graphene in HepG2 cells causes mitochondrial depolarization, leading to apoptosis.^215^ GBMs increase oxygen consumption, dissipate membranes, and activate the mitochondrial pathway, inducing apoptosis.^216^ GO also enhances electron transport activity in mitochondria, generating ROS during respiration.^217^ This results in oxidative and thermal stress, damaging mitochondrial respiration and causing toxicity. Additionally, GO accepts electrons from cellular proteins and cytochrome *C*, further contributing to apoptosis and necrosis through oxidative stress.^218^

##### 9.2.4.4 DNA damage

The high surface area, surface charge, and small size of GO can lead to genotoxicity and DNA damage, including strand breakage, chromosomal fragmentation, mutations, and oxidation of DNA adducts.^219,220^ In mice, IV injections of GO (20 mg kg^−1^) caused mutagenesis by interacting with DNA and breaking down the nuclear membrane, compared to cyclophosphamide (50 mg kg^−1^).^221,222^ The interaction between GO and DNA base pairs alters the helical axis, deforming DNA's end base pairs and potentially causing genotoxicity.^223^ Oxidative stress from GO triggers inflammation through MAPK, TGF-β, and NF-κB pathways, leading to DNA adducts, chromosomal fragmentation, and mutations. Decreased expression of CDK2 and CDK4 increases p53, Rad51, and MOGG1-1 expression, contributing to DNA damage. This can lead to cancer and may affect reproductive organs, impacting fertility and offspring health.^224^

##### 9.2.4.5 Inflammatory response

At high doses, intratracheal or IV administration of GO causes inflammatory effects, resulting in the infiltration of inflammatory cells, pulmonary edema, and granuloma formation. In the blood, platelets play a crucial role in clot formation to eliminate pathogens and particulate matter.^225^ After IV injection of GO, it directly stimulates the formation of thrombin-rich platelets, obstructing lung vessels. Subcutaneous injection of GO for 21 days increases inflammatory responses, including cytokine secretion (IL-6, IL-12, TNF-α, MCP-1, and IFN-g).^226^ GBMs stimulate Th1/Th2 inflammatory responses through chemokine, cytokine, and monocyte release.^227^ Graphene and rGO activate the NF-κB signaling pathway in cells, stimulated by IL-1 and TNF-α, moving from the cytoplasm to the nucleus *via* IκB binding and aiding cytokine synthesis. GBMs also activate the TLR4 and TLR9 pathways.^228^

##### 9.2.4.6 Apoptosis

Apoptosis, the gene-regulated process of cell destruction, occurs when GO and rGO are inhaled through the lungs in mice, leading to inflammation and apoptosis. GO and graphene physically damage cell membranes, increase membrane penetration, and alter mitochondrial potential.^229^ ROS activation influences the MAPK, TGF-β, and caspase-3 signaling pathways through mitochondrial apoptotic cascades, resulting in apoptosis. Even at low doses, rGO causes apoptosis by activating the mitochondrial membrane.^160^ GBMs exhibit different apoptotic pathways, including ROS generation through interaction with protein receptors and B-lymphocytes (Bcl-2) and transmitting apoptosis signals to the DNA nucleus *via* GO-COOH. GO-PEI damages T lymphocyte membranes, triggering the apoptosis pathway.^230^

##### 9.2.4.7 Autophagy

Cellular components undergo self-degradation, known as non-apoptotic cell death. Autophagosome components include Beclin 1, multiple autophagy-related proteins, microtubule-associated protein light chain 3 (LC3), and p62.^231^ Exposure to various nanoparticles causes autophagosome accumulation, while the autophagy process removes extracellular components and protects the organism within the cytoplasmic membrane.^232–235^ GQDs and GO induce autophagosome accumulation and the LC3-I to LC3-II conversion, inhibiting p62 protein degradation. GO also triggers TLR4 and TLR9 in colon cancer cells (CT26), a pathway related to macrophage-mediated phagocytosis.^236,237^

##### 9.2.4.8 Necrosis

This alternative form of cell death is induced by cellular injury or inflammatory responses. High-dose (50 mg mL^−1^) exposure to pristine graphene induces apoptosis and necrosis.^238^ Elevated cytoplasmic Ca^2+^ levels, LDH leakage, and mitochondrial pore permeability lead to apoptosis and necrosis. The TLR4 signaling pathway and autocrine TNF-α activation induce macrophagic necrosis. GO-CDDP triggers necrosis by increasing RIP3 and decreasing RIP1 proteins, releasing high-mobility group B1 in cytoplasmic cells.^239,240^

## 10 Concluding remarks

CNS diseases are serious and incurable health ailments with no medical cure accessible anywhere on the globe. Patients have major physiological, emotional, and social repercussions that can only be improved *via* surgical operations, pharmacological treatment, and therapy. A complete recovery is impossible to attain. The present emphasis is on the critical requirement for innovative approaches. Recent research and clinical trials addressing these challenges by studying biomaterials, neuroprotective medicines, neuromodulatory stimulation, and cell-based therapies indicate potential for reducing inflammation and improving neuroregeneration. However, optimizing these studies requires extensive epidemiological research and adaptive trial designs to address issues such as low incidence and variability. The huge potential of GBMs improves the efficacy of CNS therapies, particularly SCI therapy. However, it confronts considerable challenges, including governmental approval processes and the necessity to adapt GBMs to the SCI microenvironment. Further research should concentrate on improving GBMs performance, such as by ensuring accurate cell labeling, precise drug delivery, and interaction with scar tissue dynamics. Graphenic materials have the potential to promote neuroregeneration by sustaining viable neural networks while also boosting angiogenesis and axonal development. However, transferring these findings to human patients necessitates overcoming physiological differences while also guaranteeing biocompatibility and functioning of scaffold designs. Developing reliable correlations between scaffold features and biological responses is critical for personalized medicine methods in SCI therapy. Overall, GBMs have the potential to be multifunctional biological platforms that support a variety of SCI treatment techniques, but further research and clinical translation are required to fully realize their advantages.

## Data availability

As this manuscript is a review, no primary research results, software or code have been included and no new data were generated or analyzed as part of this review.

## Author contributions

A. Yari-Ilkhchi has conceptualized, written the original and review drafts, reviewed, and edited the manuscript. N. Hamidi has written the review, and edited the manuscript. M. Mahkam has conceptualized, supervised, reviewed, and edited the manuscript. A. Ebrahimi-Kalan has conceptualized, supervised, reviewed, and edited the manuscript. All authors have approved the final version of the text.

## Conflicts of interest

The authors declare no conflict of interest.
